# Human Liver Memory CD8^+^ T Cells Use Autophagy for Tissue Residence

**DOI:** 10.1016/j.celrep.2019.12.050

**Published:** 2020-01-21

**Authors:** Leo Swadling, Laura J. Pallett, Mariana O. Diniz, Josephine M. Baker, Oliver E. Amin, Kerstin A. Stegmann, Alice R. Burton, Nathalie M. Schmidt, Anna Jeffery-Smith, Nekisa Zakeri, Kornelija Suveizdyte, Farid Froghi, Giuseppe Fusai, William M. Rosenberg, Brian R. Davidson, Anna Schurich, A. Katharina Simon, Mala K. Maini

**Affiliations:** 1 Division of Infection and Immunity, University College London, London, UK; 2 Centre for Immunobiology, Blizard Institute, Barts and the London School of Medicine and Dentistry, QMUL, London, UK; 3 Institute for Liver and Digestive Health, University College London, London, UK; 4 Department of Infectious Diseases, Kings College London, London, UK; 5 The Kennedy Institute of Rheumatology, NDORMS, University of Oxford, Oxford, UK

**Keywords:** autophagy, T cell, liver, tissue-resident, hepatitis B virus, mitophagy, IL-15, immunometabolism

## Abstract

Tissue-resident memory T cells have critical roles in long-term pathogen and tumor immune surveillance in the liver. We investigate the role of autophagy in equipping human memory T cells to acquire tissue residence and maintain functionality in the immunosuppressive liver environment. By performing *ex vivo* staining of freshly isolated cells from human liver tissue, we find that an increased rate of basal autophagy is a hallmark of intrahepatic lymphocytes, particularly liver-resident CD8^+^ T cells. CD8^+^ T cells with increased autophagy are those best able to proliferate and mediate cytotoxicity and cytokine production. Conversely, blocking autophagy induction results in the accumulation of depolarized mitochondria, a feature of exhausted T cells. Primary hepatic stellate cells or the prototypic hepatic cytokine interleukin (IL)-15 induce autophagy in parallel with tissue-homing/retention markers. Inhibition of T cell autophagy abrogates tissue-residence programming. Thus, upregulation of autophagy adapts CD8^+^ T cells to combat mitochondrial depolarization, optimize functionality, and acquire tissue residence.

## Highlights

• An increased rate of basal autophagy is a hallmark of liver-resident CD8^+^ T cells
• Enhanced T cell autophagy can be imprinted by IL-15 or hepatic stellate cells
• Autophagy induction is required for tissue-residence programming *in vitro*
• Enhanced autophagy maintains T_RM_ mitochondrial fitness in the liver

## Graphical Abstract

**Figure:**
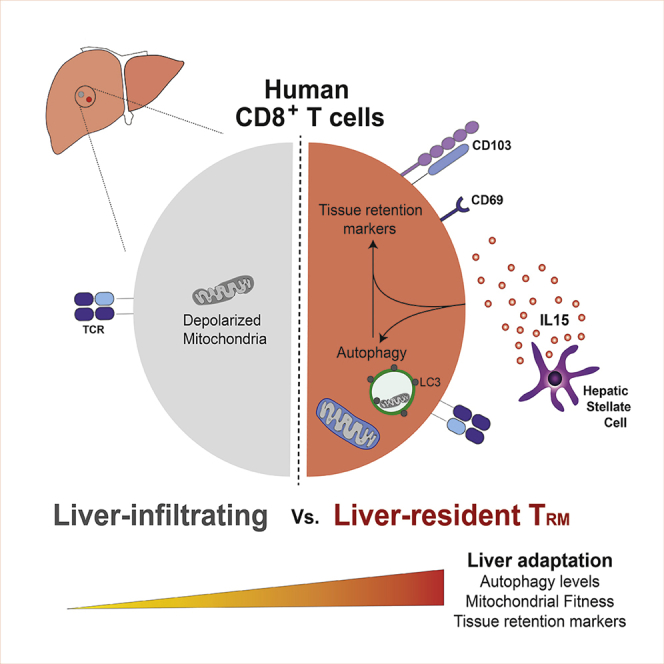

## Teaser

Swadling et al. show that an increased rate of basal autophagy is a hallmark of intrahepatic lymphocytes, particularly liver-resident CD8^+^ T cells and that *in vitro* T_RM_ programming requires autophagy induction. Upregulation of autophagy adapts CD8^+^ T cells to combat mitochondrial depolarization, optimize functionality, and acquire tissue residence.

## Introduction

The liver has a distinct tolerogenic immune environment that is exploited by hepatotropic infections and primary and metastatic tumors. With deaths from viral hepatitis now exceeding those resulting from tuberculosis and HIV (Graber-Stiehl, 2018), and primary liver cancer predicted to rise to the 13^th^ leading cause of death worldwide by 2040 (Foreman et al., 2018), it is essential that we gain a better understanding of liver immunity to aid development of immunotherapies.

T cells have an important role in clearing virus-infected and cancerous cells, but they require tight regulation to avoid immunopathology. Many T cell-tolerizing mechanisms are present in the liver (reviewed in Maini and Pallett, 2018) to avoid an excessive immune response to the constant flow of microbial products and diet-derived antigens entering the liver from the gut via the portal vein (Protzer et al., 2012). The liver is also unique in that most of its blood supply is venous and, therefore, relatively low in oxygen and flow rate when compared with arterial blood (reviewed in Carreau et al., 2011), and key T cell metabolites may be depleted within the liver microvasculature (Pallett et al., 2015, Das et al., 2008). However, little is known about the T cell-intrinsic adaptations required to survive and retain functionality in the liver.

We have recently defined a population of CD8^+^ tissue-resident memory T (T_RM_) cells, which reside in the liver without recirculating through the blood and that are preferentially expanded in patients with well-controlled hepatitis B virus (HBV) infection (Pallett et al., 2017). Liver-resident T_RM_ cells have a distinct phenotype, transcription factor expression, and the capacity to maintain efficient interleukin (IL)-2 and interferon (IFN)-γ production in the tolerogenic liver, but the cellular processes and metabolic state driving these adaptations have not been defined (Fernandez-Ruiz et al., 2016, Tse et al., 2011).

One constitutive, but highly dynamic, cellular process that all cells require and that is regulated to maintain homeostasis under cellular stress is macroautophagy (subsequently referred to here as “autophagy”). Autophagy is a highly conserved, lysosome-mediated, intracellular bulk-recycling process (Clarke and Simon, 2019). The nucleation of double-membrane vesicles, called autophagosomes, facilitates engulfment of a portion of the cytoplasm for delivery to the lysosome for degradation (Clarke and Simon, 2019). Autophagy has two main roles: first, it allows cells to maintain their cellular homeostasis by removing unwanted cytoplasmic content (pathogens, protein aggregates, damaged organelles, and reactive oxygen species [ROS]); and second, it provides biomolecules for cellular metabolism through the catabolism of proteins and complex lipids. The importance of this cellular process to diverse human T cell memory subsets, including T_RM_ cells, is not known.

Here, we address the hypothesis that liver-resident T cells use higher constitutive autophagy levels to maintain homeostasis in the liver. We characterize autophagy levels in human memory T cell subsets, including liver-resident T cells and HBV-specific T cells. Making use of our regular access to fresh human liver samples, we show that intrahepatic lymphocytes have a higher *ex vivo* level of autophagy, with lymphocytes that reside in the liver showing the highest rates of autophagy (T_RM_ cells and mucosal-associated invariant T cells [MAITs]). Recirculating T cells specific for the hepatotropic infection HBV also show high levels of autophagy. Recently activated, proliferating, or highly functional T cells have enhanced rates of autophagy, and maintenance of mitochondrial fitness is lost upon treatment with autophagy inhibitors. Finally, we show that the prototypical liver cytokine IL-15, required for the induction of liver-resident T cells, can also upregulate T cell autophagy, whereas blockade of autophagy abrogates T_RM_ cell programming of CD8^+^ T cells.

## Results

### Higher *Ex Vivo* Autophagy Levels Are Characteristic of Intrahepatic Lymphocytes

To measure autophagy in human T cells, we employed an established flow-cytometry-based assay (FlowCellect autophagy LC3 antibody-based kit, Merck Millipore/Luminex; Eng et al., 2010) that has been previously applied to human and murine lymphocyte subsets (O’Sullivan et al., 2016, Clarke et al., 2018), in particular, T cells (Puleston et al., 2014, Kabat et al., 2016, Sanderson and Simon, 2017). A reliable and specific marker of autophagic vesicles (autophagosomes) is LC3 (microtubule-associated protein 1 light chain 3)—a cytosolic protein that is lipidated and then incorporated into *de novo-*generated autophagosomes (Klionsky et al., 2016). To assay basal autophagy levels, cells are selectively permeabilized to extract cytosolic LC3-I; then autophagosome-bound LC3-II is detected using a fluorescently labeled anti-LC3 antibody (“unblocked”; see Method Details). We also measured the accumulation of autophagosomes over time by inhibiting autophagosome breakdown with bafilomycin A1 (bafA1), an endosomal acidification inhibitor (Klionsky et al., 2016; “blocked data”). Finally, as a proxy for autophagic flux and to eliminate the variation in the number of autophagosomes at baseline (including tissue-specific variation and cell size), we calculated the ratio of LC3 staining for blocked versus unblocked samples.

**Figure 1:**
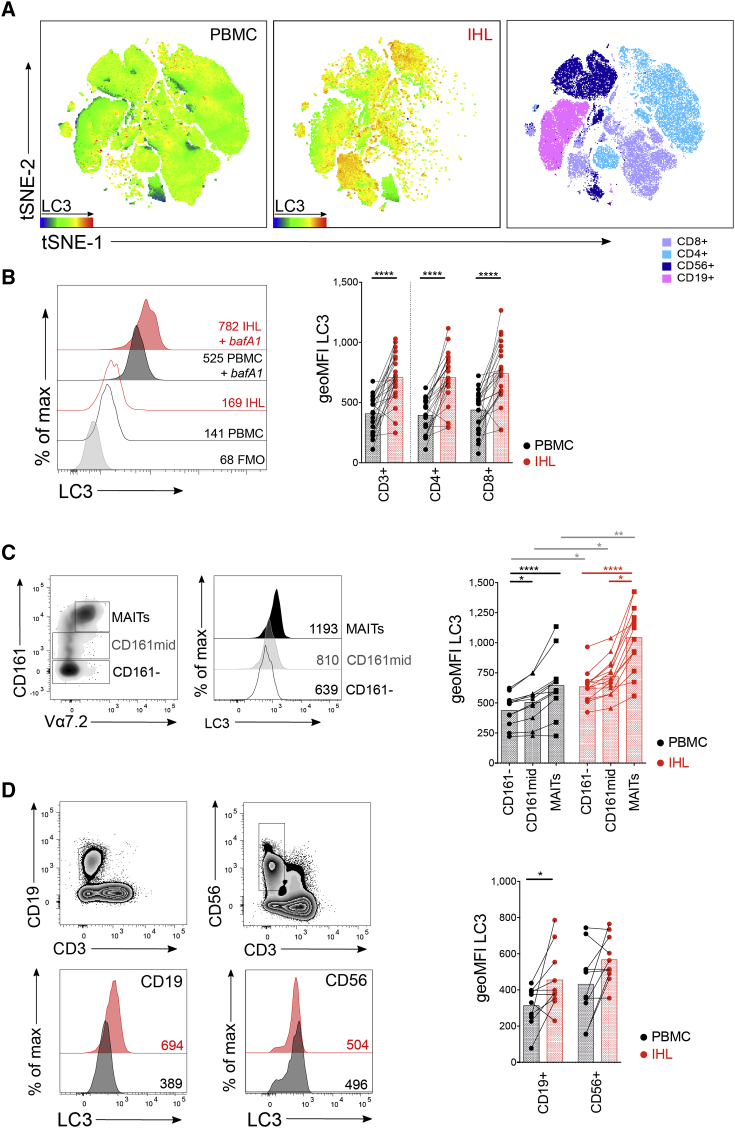
Intrahepatic lymphocytes Are Characterized by High *Ex Vivo* Autophagy Levels (A) The dimensionality reduction algorithm tSNE was applied to flow cytometry data (single cell expression values from total live CD45^+^ singlet lymphocytes for: CD3, CD4, CD8α, CD19, CD103, CD69, pan-ɣδ T cell receptor (TCR), pan-αβ TCR, CD161, CD56, and LC3) to generate a two-dimensional map of lymphocytes from paired PBMC (left) and IHL (middle) samples from two individuals colored by intensity of LC3 or by lymphocyte subset (right; example gating Figure S1A). (B) Histograms (gated on CD8^+^; ± bafilomycin A1 [bafA1] treatment, 0.1 μM; FMO for LC3) and summary data for LC3 staining of paired peripheral (PBMCs; black) and intrahepatic (IHLs; red) T cells (23 biological replicates). (C and D) Example of gating, histograms, and summary data for LC3 staining of CD161^−^, CD161^mid^, and mucosal-associated invariant T cells (MAITs; CD161^hi^ Vα7.2^+^; 11–14 biological replicates) (C) and CD19^+^ (B cells) and CD56^+^ (NK cells) lymphocytes (10 biological replicates) (D). Cells were treated with bafA1 unless otherwise stated (unblocked data in Figure S1) (A, C, and D). Wilcoxon paired t test (B and D). For pairwise multiple comparisons (within PBMC/IHL comparisons) Friedman test (ANOVA) with Dunn’s post hoc test (C). For multiple unpaired comparisons (between PBMC and IHL for a given subset) Kruskal-Wallis (ANOVA) with Dunn’s post hoc test. Bars at mean (B, C, and D). ^∗^p < 0.05, ^∗∗^p < 0.005, ^∗∗∗∗^p < 0.0001.

We first investigated whether lymphocytes that have been exposed to the tolerogenic liver microenvironment display a different level of autophagy to peripheral lymphocytes isolated from blood. To do so, we assayed the level of autophagy in human intrahepatic lymphocytes (IHLs) from explanted liver samples, liver perfusates, and paired blood (peripheral blood mononuclear cells [PBMCs]). To observe the relative autophagy levels of all lymphocyte subsets from paired PBMC and IHL samples, we performed dimension reduction by tSNE (t-distributed stochastic neighbor embedding) on multiparametric flow cytometry data to visualize the relative LC3 intensity of each cell. PBMCs and IHLs showed overlapping distributions, but overall, the intensity of LC3 staining was greater on IHLs (Figure 1A; gating and example plots in Figure S1A). Several clusters of lymphocytes with high-intensity LC3 staining were uniquely observed in IHL samples. We, therefore, extended this analysis to a larger cohort of paired human blood and liver samples. Intrahepatic T cells had a significantly higher *ex vivo* level of autophagy than T cells isolated from blood when gating on CD4^+^, CD8^+^, or total CD3^+^ T cells (Figure 1B; unblocked data [no bafA1] and blocked/unblocked ratio in Figures S1B and S1C, respectively). Although autophagy levels increased with T cell granularity (SSC [side scatter]), they did not directly correlate with T cell size (FSC [forward scatter]), and enhanced *ex vivo* autophagy levels were not higher because of T cells showing a different morphology in the liver (Figure S1A). Differences in autophagy levels between blood and liver were also not attributable to differences in sample processing because they were maintained when IHLs isolated from perfusion fluid of healthy transplant livers, which are processed identically to blood, were used (Figure S1D).

MAITs (CD161^hi^ Vα7.2^+^), a population of T cells that has recently been shown to reside long term in the liver (Salou et al., 2019), also had higher levels of autophagy when compared with CD161^mid^ or CD161^−^ T cells in the blood or liver, with each of these subsets having higher LC3 levels in the liver than their circulating counterparts (Figure 1C). Other lymphocytes assayed also had a higher level of autophagy in the liver when compared with the equivalent population in the blood, including CD19^+^ B cells (Figure 1D). CD56^+^ natural killer (NK) cells were the exception, where autophagy levels were comparable between peripheral and intrahepatic populations (Figure 1D). Overall, several lymphocyte subsets isolated from the liver had a higher level of autophagy than their counterparts isolated from blood, in particular, T cells.

### Enhanced Autophagy by Intrahepatic T Cells Is Not a Result of a Difference in T Cell Memory Subset Frequency or Recent Proliferation

**Figure 2:**
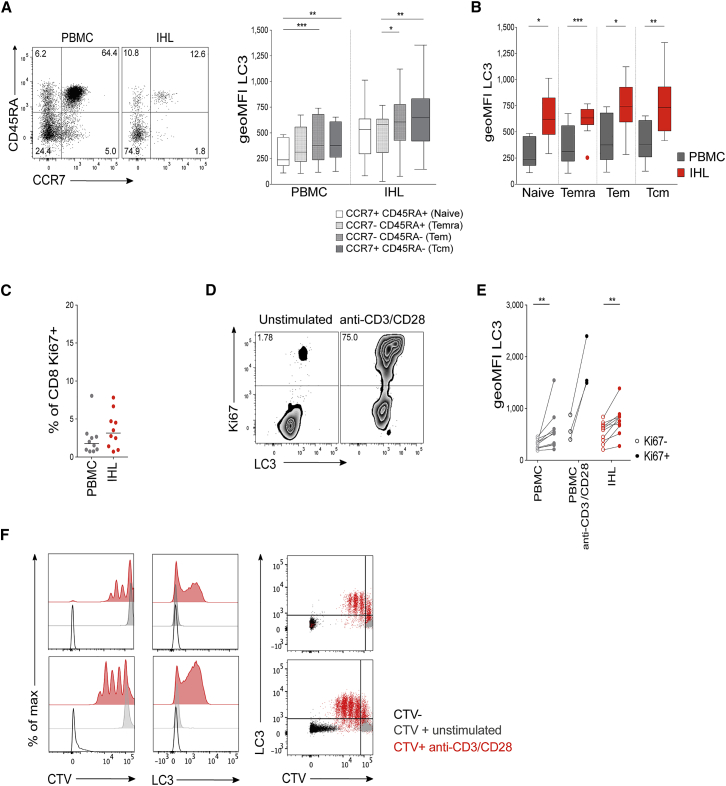
High Autophagy Level of Intrahepatic T Cells Is Not a Result of a Difference in Differentiation Status or Recent Proliferation (A) Example plot of CD45RA versus CCR7 staining (CD8^+^ T cells) from a PBMC or IHL sample and summary data for LC3 staining of CD8^+^ T cell memory subsets (PBMC, 9; and IHL, 15, biological replicates; box whisker, Tukey). (B) Comparison of LC3 staining of CD8^+^ T cell memory subsets between paired PBMC and IHL samples (9 biological replicates; box whisker, Tukey; outliers shown as dots). (C) *Ex vivo* CD8^+^ T cell Ki67 expression. (D and E) Example plots (CD8^+^ T cells, PBMC) (D) and summary data for LC3 staining on Ki67^−^ and Ki67^+^ CD8^+^ T cells (E) in PBMCs and IHLs *ex vivo* (10 biological replicates) or after anti-CD3/CD28 stimulation (overnight, 0.5 μg/mL each; three biological replicates) in PBMCs. (F) Histograms showing the dilution of CellTrace Violet (CTV), LC3 staining, and co-staining of LC3 and CTV on CD8^+^ T cells after 5 days of stimulation with anti-CD3/CD28 (red), compared with that without stimulation (gray) or without CTV staining (black; two representative biological replicates of five, PBMCs). Cells were treated with bafA1 (A–F). Friedman test (ANOVA) with Dunn’s post hoc test for pairwise multiple comparisons (A and B). Mann-Whitney t test (C and E). ^∗^p < 0.05, ^∗∗^p < 0.005, ^∗∗∗^p < 0.001.

We investigated whether the difference in T cell autophagy levels in the liver and blood was due to a different T cell subset composition in the two compartments. In both IHLs and PBMCs, CD8^+^ T cells with an effector memory (T_EM_, CCR7^−^CD45RA^−^) or central memory (T_CM_; CCR7^+^CD45RA^−^) phenotype had the highest level of autophagy, with naive (CCR7^+^CD45RA^+^) and terminally differentiated effector memory (T_EMRA_; CCR7^−^CD45RA^+^; Sallusto et al., 1999) T cells showing lower levels (Figure 2A). The liver is enriched for memory T cells and has few naive T cells relative to blood (Pallett et al., 2017); however, all T cell subsets had a higher level of autophagy in the liver, indicating that differences between these compartments were not purely due to a different balance of T cell memory subsets (Figure 2B).

Autophagy is enhanced when murine (Hubbard et al., 2010, Kabat et al., 2016, Watanabe et al., 2014, Botbol et al., 2015, Yang et al., 2013) and human T cells are activated and proliferating (Watanabe et al., 2014). If the liver housed a larger proportion of proliferating T cells, this may explain their higher autophagy levels. The percentage of CD8^+^ T cells expressing Ki67, a marker of recent cell cycling, was not significantly higher on T cells within the liver than it was in the circulation (Figure 2C; Pallett et al., 2017). We confirmed in humans that *ex vivo* proliferating (Ki67^+^) CD8^+^ T cells in both blood and liver had higher autophagy levels than Ki67^−^ CD8^+^ T cells (Figures 2D and 2E) and that recently activated (Figure 2E) and dividing human CD8^+^ T cells (Figure 2F) showed upregulated autophagy, as has been shown previously in mice (Hubbard et al., 2010, Pua et al., 2007). We conclude that T cells in the liver show enhanced autophagy levels relative to their counterparts in the blood, which cannot be accounted for solely by their differentiation or proliferation status.

### Autophagy Levels Are Highest in T Cells That Reside in the Liver

We have recently defined a population of CD8^+^ T cells that are retained and maintain functionality in the immunosuppressive liver environment (Pallett et al., 2017). These liver-resident CD8^+^ T cells are distinguished by expression of the tissue-retention marker CD69 (a sphingosine 1-phosophate receptor-1 antagonist), with or without CD103 (integrin-αE, which in combination with integrin-β7 can bind to E-cadherin on endothelial cells and hepatocytes). CD69^+^CD103^+/−^ T_RM_ cells are enriched in the liver, with CD69^+^CD103^+^ being completely absent from the blood (example plot in Figure S1A; Pallett et al., 2017, Stelma et al., 2017).

**Figure 3:**
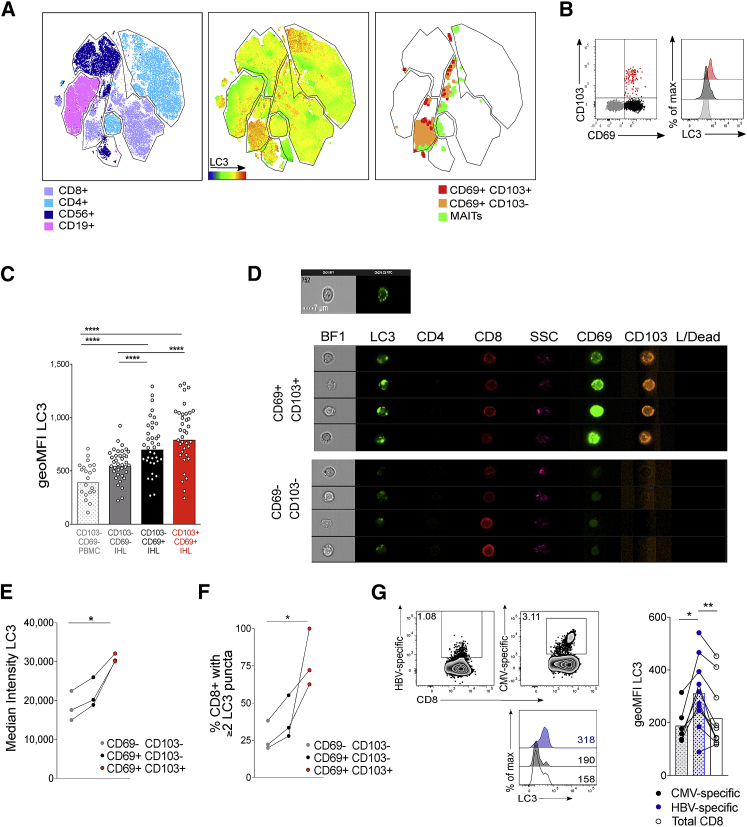
Autophagy Levels Are Highest in T Cells that Reside in the Liver (A) tSNE was applied to flow cytometry data (single-cell expression values from total live CD45^+^ singlet lymphocytes for CD3, CD4, CD8⍺, CD19, CD103, CD69, pan-ɣδ TCR, pan-αβ TCR, CD161, CD56, and LC3) to generate a two-dimensional map of lymphocytes from paired PBMC (left) and IHL (middle) samples from two individuals. Cells are colored by lymphocyte subset (left; example gating in Figure S1A) and by intensity of LC3 staining for PBMCs and IHLs combined (middle). CD8^+^ T_RM_ cells (pan-αβ TCR^+^CD3^+^CD8^+^CD69^+^CD103^+/−^) and MAITs (CD3^+^CD161^hi^TCR-Vα7.2^+^) are plotted (right). (B and C) Representative plots (B) and cumulative data (C) of LC3 staining on liver-resident (CD69^+^CD103^−^ [black] and CD69^+^CD103^+^ [red] subsets) and non-resident liver infiltrating T cells (CD69^−^CD103^−^ [gray]) in the human liver or CD69^−^CD103^−^ T cells in the blood (white). Bars at geometric mean (see also Figure S2; PBMC, 21; and IHL, 38, biological replicates). (D) Example images of single liver-resident (CD69^+^CD103^+^) or recirculating (CD69^−^CD103^−^) CD8^+^ T cells from a human perfusate sample by ImageStream (see also Figure S3; representative of three biological replicates). L/dead, fixable live dead. (E) Mean intensity of LC3 staining of T_RM_ cells and recirculating intrahepatic CD8^+^ T cells by ImageStream. (F) The percentage of T_RM_ cells and recirculating intrahepatic CD8^+^ T cells that contained two or more LC3 puncta by ImageStream. (G) Representative *ex vivo* dextramer staining, histograms of LC3 staining, and summary data for LC3 staining of HBV-specific (blue; see Method Details for panel of HBV dextramers targeting HBV core, surface, and polymerase), CMV-specific (black; pp65485-504 HLA-A^∗^02, NLVPMVATV) and total CD8^+^ T cells (white), in PBMCs from patients chronically infected with HBV (Table S1; 10 biological replicates). Representative examples from one of two technical replicates (E and F). Cells treated with bafA1 (unblocked data in Figure S2) (A–G). Kruskal-Wallis (ANOVA) with Dunn’s post hoc test for multiple unpaired comparisons (C). Friedman test (ANOVA) with Dunn’s post hoc test multiple paired comparisons (C, E, and F). Wilcoxon t test (G). ^∗^p < 0.05, ^∗∗^p < 0.005, ^∗∗∗∗^p < 0.0001.

We assessed the rate of autophagy within the liver-resident CD8^+^ T_RM_ compared with non-resident, liver-infiltrating T cells (CD69^−^CD103^−^) transiting through the liver at the time of sampling. By looking more closely at the subpopulations of CD8^+^ T cells in the tSNE output, we observed that CD69^+^CD103^+/−^ CD8^+^ T_RM_ cells clustered in areas of high-LC3 expression, relative to all PBMCs and IHLs (Figure 3A). Extending those data to a cohort of 38 paired human blood and liver samples, a stepwise increase in autophagy levels was seen from CD69^−^CD103^−^ liver-infiltrating, to CD69^+^CD103^−^, to CD69^+^CD103^+^ liver-resident memory CD8^+^ T cells, suggesting that rates of autophagy are greatest in populations of T cells that reside long term in the liver (Figures 3B and 3C; ratio blocked versus unblocked in Figure S2A).

We next tested whether the *ex vivo* difference in autophagy rates in human liver-resident memory CD8^+^ T cells could be validated by two further markers of autophagosomes and by imaging flow cytometry. We found that CD8^+^ T_RM_ cells are also characterized by higher autophagy levels when quantifying the relative accumulation of the autophagosome-associated cargo protein sequestosome-1 (p62, SQSTM1; Figure S2B) and the use of the autophagic vesicle-specific dye Cyto-ID (Figures S2C and S2D; example plot in Figure S1A). To directly visualize autophagosomes within T cells, we then used a recently developed autophagy assay employing imaging flow cytometry (ImageStream; Klionsky et al., 2016, Phadwal et al., 2012, Puleston et al., 2014) to visualize LC3 within autophagosome puncta (gating strategy and staining controls in Figure S3). We confirmed by ImageStream that there was higher per-cell median fluorescence for LC3, representing a higher total number of autophagosomes (example cell images in Figure 3D), for liver-resident T cells relative to peripheral CD8^+^ T cells or liver-infiltrating T cells (Figure 3E). We then quantified distinct autophagosomes as LC3^+^ puncta within T cells and showed that most T_RM_ cells contained multiple autophagosome puncta, whereas recirculating CD8^+^ in the liver mainly contained one or no autophagosomes (Figure 3F). Taken together, these data, based on four different approaches, showed enhanced autophagy levels in CD8^+^ T cells that are resident versus those infiltrating the liver or in the periphery.

Using PBMCs isolated from patients chronically infected with HBV (Table S1), we next asked whether a population of memory T cells that has been primed and/or encountered cognate antigen within the liver would also be imprinted with enhanced autophagy levels. We stained PBMCs with major histocompatibility complex (MHC)-class I dextramers loaded with peptides corresponding to immunodominant HBV and cytomegalovirus (CMV) epitopes in HLA-A^∗^02^+^ patients and compared the autophagy levels of total CD8^+^ T cells, HBV-specific T cells, and a population of memory T cells directed against a control virus (CMV specific). HBV-specific T cells showed a higher level of autophagy than total CD8^+^ T cells or CMV-specific T cells assayed in the same patients (Figure 3G). The small population of HBV-specific memory T cells with high levels of autophagy that persists in patients chronically infected with HBV may represent a pool of T cells that have enhanced autophagy levels imprinted on them when in the liver. Taken together, these data show that autophagy levels are highest in T cell subsets resident or encountering antigens within the liver.

### Enhanced Autophagy Levels Are Associated with Enhanced Effector Function and Mitochondrial Fitness in T Cells

We next investigated the functional consequence of enhanced autophagy for human CD8^+^ T cells to determine whether the T cells that gain specific effector functions on activation are also those that have the highest induction of autophagy.

**Figure 4:**
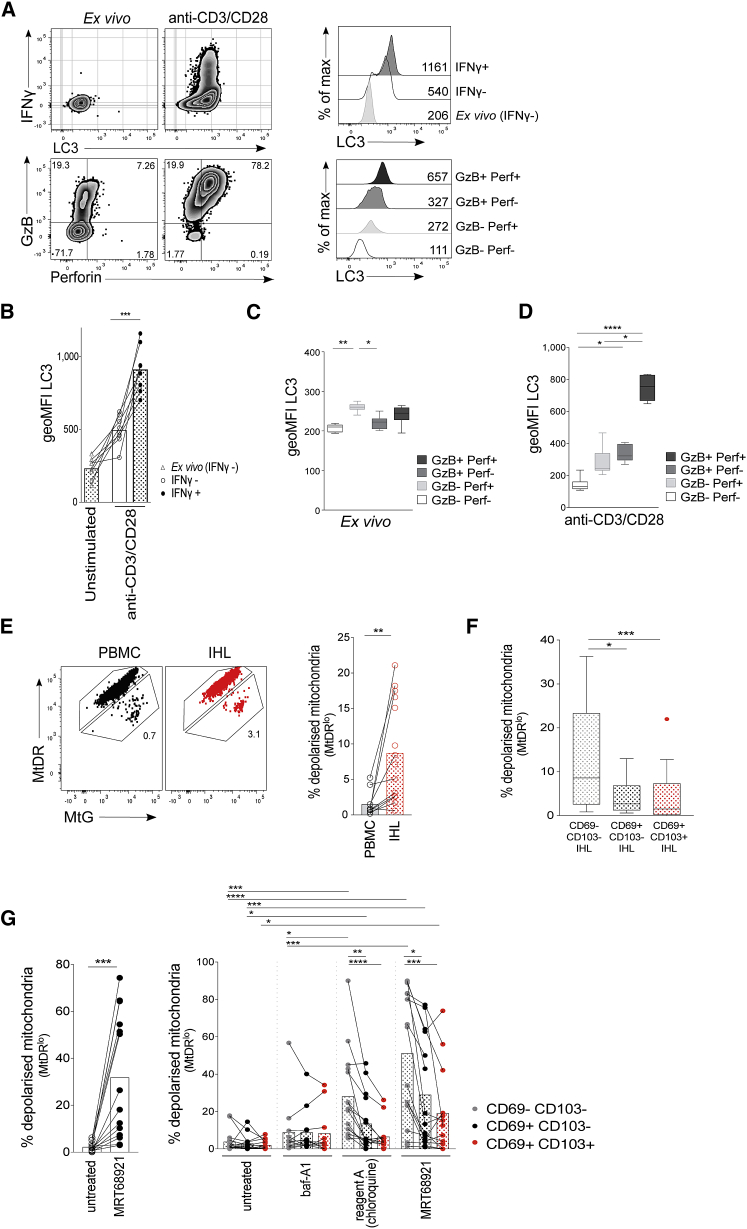
Enhanced Autophagy Levels Are Linked to Effector Function and Mitochondrial Fitness in Human T Cells (A) Example plots of IFN-γ, LC3, granzyme B (GzB), and perforin (perf; gated on CD8^+^ T cells) and histograms of LC3 staining for PBMC *ex vivo* or after anti-CD3/CD28 stimulation (3 days; see also Figure S3). (B) LC3 staining of CD8^+^ T cells from unstimulated PBMCs (IFN-γ^−^), IFN-γ^−^ and IFN-γ^+^ CD8^+^ T cells after anti-CD3/CD28 stimulation (3 days; eight biological replicates). (C and D) LC3 staining on GzB and perf-expressing CD8^+^ T cells *ex vivo* (C) and after anti-CD3/CD28 stimulation (D) (3 days; eight biological replicates; box whisker, Tukey). (E) Example mitochondrial staining of CD8^+^ T cells in blood (PBMCs; black) and liver (IHLs; red) and summary data for the *ex vivo* percentage of total CD8^+^ T cells with depolarized mitochondria (mitoTracker deep red [MtDR]^lo^; see also Figure S4; PBMCs, 10; and IHLs, 15 biological replicates). (F) *Ex vivo* percentage of CD8^+^ T_RM_ cell subsets in the liver with depolarized mitochondria (14 biological replicates; box whisker, Tukey; outliers shown as dots). (G) The percentage of total CD8^+^ T cells or CD8^+^ T_RM_ cell subsets with depolarized mitochondria after overnight culture of IHLs with DMSO (untreated), MRT68921 dihydrochloride (10 μM), bafA1 (0.1 μM), or reagent A (chloroquine diphosphate, 1:1000, FlowCellect LC3 kit; 13–15 biological replicates). Cells were treated with bafA1 (A–D). Bars at mean (B, E, and G). Friedman test (ANOVA) with Dunn’s post hoc test for pairwise multiple comparisons (B, C, D, and F). Kruskal-Wallis test with Dunn’s post hoc test for unpaired multiple comparisons (G). Mann-Whitney unpaired t test for total CD8^+^ PBMCs versus IHLs (E). Wilcoxon paired t test for untreated versus that treated with MRT68921 (G). Bars at mean (E and G). ^∗^p < 0.05, ^∗∗^p < 0.005, ^∗∗∗^p < 0.001, ^∗∗∗∗^p < 0.0001.

Upon *in vitro* activation, CD8^+^ T cells that produced IFN-γ also showed an enhanced rate of autophagy (Figures 4A and 4B; LC3 staining and cytokine release controls are shown in Figure S3E; LC3 staining by FlowCellect kit was performed in parallel to brefeldin A treatment and permeabilization with a transcription factor staining kit to confirm that autophagosome quantitation was equivalent by intracellular staining, r = 0.9404 p < 0.0001 for CD8^+^ T cells; Figure S3F). Without stimulation, T cells with cytolytic potential *ex vivo* also had higher autophagy levels than did T cells that lacked perforin or granzyme B (GzB) expression (Figure 4C). When stimulated, the expanded population of CD8^+^ T cells expressing cytolytic markers showed an increase in autophagy, and T cells that co-expressed GzB and perforin were those with the highest autophagy levels (Figure 4D). There is, therefore, an association on a per-cell basis between the upregulation of autophagy by human CD8^+^ T cells upon activation and the acquisition of T cell effector functions.

Murine T cells lacking autophagy because of ATG7 deletion have been shown to accumulate mitochondrial mass and ROS (Puleston et al., 2014); a higher level of autophagy promotes removal of depolarized mitochondria and ROS in T cells (mitophagy) (Schlie et al., 2015, Jia and He, 2011, Pua et al., 2009, Stephenson et al., 2009, Hubbard et al., 2010). This may be particularly important in an oxygen-deprived environment, such as the liver, in which mitochondrial damage and ROS accumulation can be high, as has been shown for HBV-specific T cells (Fisicaro et al., 2017, Schurich et al., 2016).

To assess mitochondrial fitness, we co-stained T cells with MitoTracker Deep Red (MtDR), a polarization-sensitive dye that only stains functional polarized mitochondria, and MitoTracker green (MtG), which stains all mitochondria (Puleston, 2015, Zinser et al., 2018). Dying T cells in the liver and blood (defined as fixable live/dead^+^) were almost exclusively MtDR^lo^, consistent with the high rates of depolarized mitochondria expected in this population (Figure S4A). Within the live lymphocyte gate, a larger proportion of total CD8^+^ T cells had depolarized mitochondria in the liver than in the blood (Figure 4E). When dissected by expression of tissue-residency markers, the liver-infiltrating CD69^−^CD103^−^ fraction had the greatest burden of dysfunctional mitochondria (Figure 4F), in line with their lower autophagy rate compared with T_RM_ cells. When autophagy was blocked overnight with the autophagy-specific inhibitor MRT68921 dihydrochloride (inhibitor of Unc-51-like autophagy-activating kinase, blocking autophagosome nucleation; Petherick et al., 2015), all liver T cell subsets accumulated depolarized mitochondria, underscoring the importance of this pathway for removal of damaged mitochondria, as has been shown for murine T cells (Figure 4G; Pua et al., 2009, Watanabe et al., 2014, Stephenson et al., 2009). Enhanced accumulation of depolarized mitochondria was also seen when using endosomal-acidification inhibitors to block autophagosomal digestion (bafA1 or reagent A, FlowCellect Kit; Figure 4G). Consistent with their lower baseline autophagy levels, with each autophagy inhibitor it was again the infiltrating, rather than resident, fraction of intrahepatic CD8^+^ T cells that accumulated the highest levels of depolarized mitochondria (Figure 4G).

One potential caveat to these findings is that a subpopulation of CD8^+^ T_RM_ cells with the ability to efflux fluorescent dyes, such as MtG, has been previously described in the human lung, spleen, and bone marrow (Kumar et al., 2018). We found that efflux^+^ T cells are also present in the human liver and are enriched within the CD69^+^CD103^+/−^ T_RM_ cell fractions (Figure S4B). However, when efflux pump inhibitors verapamil or cyclosporin A (CSA) were used, the percentage of depolarized mitochondria (MtDR^lo^) did not change (Figure S4C), showing that efflux^+^ T_RM_ cells were retained within the MtDR^+^ gate, independent of dye efflux. Therefore, the differences in depolarized mitochondria that we observed were not simply due to differential dye efflux. The accumulation of depolarized mitochondria can also be a general feature of apoptosis; however, the viability of lymphocytes treated with autophagy inhibitors overnight was not greatly reduced relative to untreated cells (Figure S5). This suggests that the accumulation of depolarized mitochondria was a consequence of a reduction in mitophagy and not secondary to any toxicity of the autophagy inhibitors.

Overall, these data suggest that the liver is an environment in which mitochondrial damage is high, and where persistent high levels of mitophagy may be required for T cells to adapt to residency within this milieu.

### *In Vitro-*Induced T_RM_ Cells Have a High Level of Autophagy, and T_RM_ Cell Induction Is Limited When Autophagy Is Blocked

**Figure 5:**
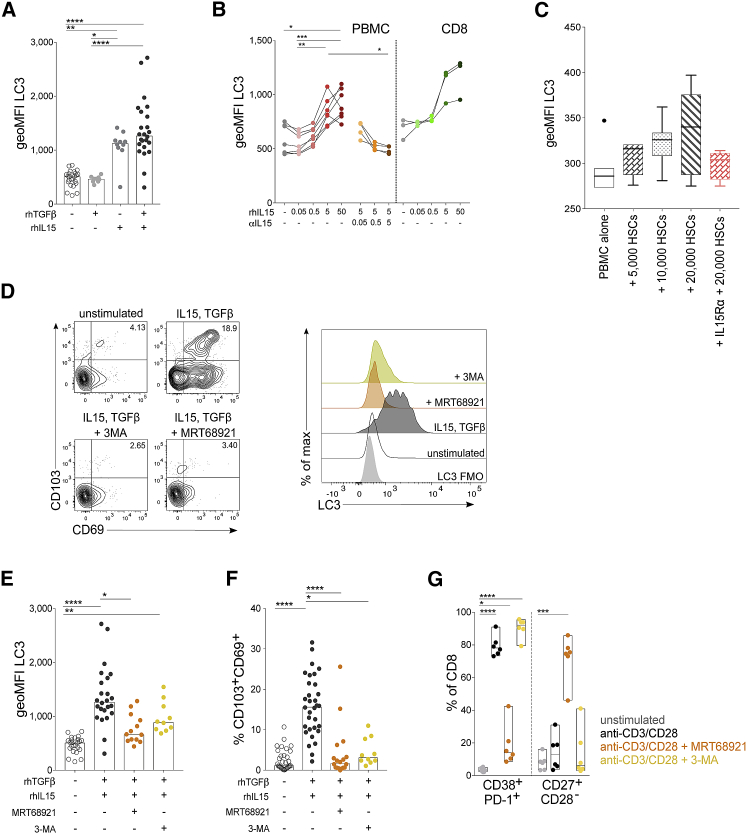
*De Novo-*Induced T_RM_ Cells Have a High Level of Autophagy, and T_RM_ Cell Induction Is Abrogated When Autophagy Is Inhibited (A) LC3 staining of CD8^+^ T cells after 6 day of PBMC culture with the following cytokines: recombinant human TGF-β (rhTGF-β, 50 ng/ml) at day 0, rhIL-15 (50 ng/mL) at day 0, sequential rhIL-15 at day 0 then rhTGF-β at day 3 (bars at median; 9–24 biological replicates). (B) LC3 staining of CD8^+^ T cells after 3 days of PBMC culture or isolated CD8^+^ T cells with rhIL-15 alone (0.05-50 ng/mL) or with anti-IL-15 blocking antibody (0.05-5 μg/mL; three to seven biological replicates). (C) LC3 staining of CD8^+^ T cells after 3 days of PBMC co-culture with isolated primary human hepatic stellate cells (pHSCs; seven biological replicates) and in the presence of IL-15 blocking with rhIL-15Rα-Fc chimera (0.01 μg/mL; four biological replicates). Box whisker, Tukey; outliers shown as dots. (D) Example plots of T_RM_ cell induction and histograms of LC3 staining after 6 days of culture with sequential rhIL-15 at day 0; then, rhTGF-β at day 3 with and without autophagy inhibitors: MRT68921 dihydrochloride (1 μM) and 3-MA (3-methyladenine; 0.5 mM). (E and F) LC3 staining (E) and magnitude of the induced CD69^+^CD103^+^ T_RM_ cell population (F) as a percentage of CD8^+^ T cells after 6 days of culture with and without autophagy inhibitors (10–32 biological replicates). (G) Phenotypic changes in total CD8^+^ T cells after stimulation with anti-CD3/CD28 (3 days) with and without the autophagy inhibitors (six biological replicates). Cells were treated with bafA1 (A, B, and D–G). Kruskal-Wallis (ANOVA) with Dunn’s post hoc test or multiple unpaired comparisons (A, E, and F). Friedman test (ANOVA) with Dunn’s post hoc test for pairwise multiple comparisons (B and C). One-way ANOVA with Holm-Sidak post hoc test multiple paired comparisons unstimulated versus other treatments (G). ^∗^p < 0.05, ^∗∗^p < 0.005, ^∗∗∗^p < 0.001, ^∗∗∗∗^p < 0.0001.

Tissue-resident T cells have only recently been recognized, and the mechanism by which residency is imprinted on T cells *in vivo* has yet to be fully decoded. We and others have previously tested a range of cytokines and T cell stimulations and identified an efficient protocol to induce CD8^+^ T_RM_ cell phenotype T cells *in vitro* from human PBMCs using sequential exposure to IL-15 and then TGF-β (Pallett et al., 2017, Sowell et al., 2017, Mackay et al., 2015), two prototypical liver cytokines. These *de novo* “induced residency” CD8^+^ T_RM_ cells express both CD69 and CD103 and have several other characteristics of human liver-resident T cells (e.g., expression of CXCR3, CXCR6, and a Blimp^hi^Eomes^lo^ phenotype; Pallett et al., 2017). Here, we used this protocol to assess whether *in vitro-*induced residency is also accompanied by enhanced levels of autophagy, consistent with T_RM_ cell staining *ex vivo*. Sequential exposure to IL-15 and TGF-β, shown to optimally induce *de novo* CD8^+^ CD69^+^CD103^+^ T_RM_ cells from PBMCs (Pallett et al., 2017) was also able to efficiently upregulate autophagy, with the latter effect attributable to IL-15 (Figures 5A and 5B). We confirmed that IL-15 alone induced autophagy in a dose-dependent manner in CD8^+^ T cells either within PBMCs or after isolation; this autophagy induction could be blocked with an anti-IL-15 monoclonal antibody (Figure 5B).

IL-15 is constitutively expressed by several liver-resident cell types, including hepatic stellate cells (HSCs) (Golden-Mason et al., 2004, Winau et al., 2007, Zhou et al., 2018) and liver macrophage populations (Golden-Mason et al., 2004). To test whether a liver-resident cell population known to produce IL-15 could recapitulate the high levels of T cell autophagy observed in liver T_RM_ cells, we co-cultured peripheral T cells with primary HSCs isolated from the healthy margins of liver resections. A trend toward a dose-dependent induction of T cell autophagy was observed after incubation of T cells with HSCs, and this induction could be partially blocked by the addition of recombinant human IL-15Rα (Figure 5C), supporting a potential role for HSC-derived IL-15 in driving the high T cell autophagy we observed in human IHLs.

To investigate whether the induction of autophagy was a prerequisite for the acquisition of a T_RM_ cell profile, we used inhibitors of autophagy during the cytokine T_RM_ cell induction protocol. The autophagy inhibitors MRT69821 dihydrochloride and 3-MA (3-methyladenine; types I and III phosphatidylinositol 3-kinases inhibitor; Mocholi et al., 2018) were able to partially block the induction of autophagy seen during *in vitro* T_RM_ cell induction (Figures 5D and 5E). Crucially, the reduction in autophagy achieved with these inhibitors abrogated the acquisition of molecules critical for tissue retention (Figures 5F and S5D).

The observed abrogation of T_RM_ cell induction was not attributable to autophagy inhibitors rendering T cells completely unresponsive to stimulation or to a loss of T cell viability. Inhibition of autophagy selectively prevented *in vitro* T_RM_ cell induction by IL-15/TGF-β without causing a significant reduction in the number of liver-infiltrating CD69^−^CD103^−^ T cells (Figure S5D) or in T_RM_ cell viability (Figure S5E). T cells could still become activated (co-expression of CD38 and PD-1) and differentiate into effectors (CD27^+^CD28^−^) when T cell receptor (TCR)-stimulated in the presence of autophagy inhibitors (Figure 5G). Overall, these data suggest that IL-15-driven induction of T cell autophagy may be specifically required for tissue-residency programming.

## Discussion

Using high-dimensional flow and imaging cytometry applied to fresh human liver samples, we show a link between greater use of autophagy and tissue-resident memory. We show that enhanced autophagy is required for optimal acquisition of tissue residency *in vitro* and for maintenance of T cell mitochondrial fitness, and may be imprinted on liver-resident CD8^+^ T cells by the prototypic hepatic cytokine IL-15.

Autophagy has an active role in the functionality and differentiation of diverse lymphocyte subsets, in particular, for long-lived and quiescent cells (Riffelmacher et al., 2018, Clarke et al., 2018, Zhu et al., 2018). Complete ablation of autophagy in total murine T cells results in defective homeostasis or responsiveness to stimulation (Schlie et al., 2015, Puleston et al., 2014, Stephenson et al., 2009, Pua et al., 2009, Jia and He, 2011, Hubbard et al., 2010). In particular, autophagy-deficient memory T cells are depleted, either because they fail to survive the transition from effector to memory T cells or because they need autophagy for their maintenance (Schlie et al., 2015, Xu et al., 2014, Puleston et al., 2014, Murera et al., 2018, DeVorkin et al., 2019); however, how varied levels of autophagy affect T cell survival, functionality, and differentiation and the importance of autophagy for human T cell subsets, in particular tissue-resident CD8^+^ T cells had not, to our knowledge, been considered.

Not only were autophagy levels high in total CD3^+^, CD4^+^, or CD8^+^ T cells isolated from human liver samples, but within the intrahepatic compartment, the T cells with the highest level of autophagy were those that expressed the tissue-retention markers CD69^+^ and CD103^+^ (Pallett et al., 2017). We demonstrated that differences between T cells in the liver and blood were not solely due to differences in intrahepatic T cell morphology, memory subset composition, or levels of activation and proliferation.

Conceptually, how might human T cells in the liver benefit from enhanced autophagy levels? We first tried to address this by asking whether the levels of autophagy in T cells were linked to their functional profile, as has been suggested in mice. In mice, autophagy may be transiently reduced immediately after T cell activation, but effector T cells show a higher level of autophagic flux than do naive T cells, and complete abrogation of autophagy has a profound effect on T cell survival and transitioning to long-lived memory T cells (Xu et al., 2014). We confirmed that autophagy was increased in effector T cells 3–5 days after TCR-mediated activation and further demonstrated that on a per-cell basis, the acquisition of T cell effector function only occurred in accordance with increased rates of autophagy.

*Ex vivo* expression of cytolytic mediators or the proliferation marker Ki67 correlated with higher levels of autophagy. Higher levels of autophagy may, therefore, be required to allow a T cell to respond to stimulation or to switch on and retain effector functions. This is supported by murine data showing that anergy is induced in CD4^+^ T cells if autophagy is inhibited at the time of TCR-mediated stimulation (Mocholi et al., 2018). This was also the case in our *in vitro* T_RM_ cell-induction model, which showed that the pro-proliferative cytokine IL-15 drives CD8^+^ T cell autophagy in a dose-dependent manner.

Autophagy is the main mechanism by which damaged, depolarized mitochondria are selectively targeted to the lysosome for their removal (mitophagy; Villa et al., 2018), and this pathway has been shown to be important in neutrophils (Riffelmacher et al., 2018), B cells (Clarke et al., 2018), and global murine T cells (Pua et al., 2009, Stephenson et al., 2009, Jia and He, 2011, Murera et al., 2018). T cell-specific autophagy knockout models uniformly demonstrate an increase in depolarized mitochondria and ROS, leading to increased T cell death (Murera et al., 2018, Jia and He, 2011, Pua et al., 2009, Stephenson et al., 2009, Watanabe et al., 2014, Schlie et al., 2015). Mitochondria can become damaged when defective mitochondrial proteins or ROS accumulate (reviewed in Haynes et al., 2013). The liver is unique in that it receives most of its blood as deoxygenated venous blood (Carreau et al., 2011) and is characterized by high levels of oxidative stress (Huang et al., 2017, García-Ruiz and Fernández-Checa, 2018). Of note, mitochondrial defects and reduced survival are also a characteristic of autophagy-deficient hepatic stellate cells (Hernández-Gea et al., 2012).

Our study showed an accumulation of depolarized mitochondria in intrahepatic T cells *ex vivo*, in keeping with the high oxidative stress milieu. Pharmacological inhibition of autophagy in human T cells can also lead to an accumulation of damaged mitochondria, suggesting a role for mitophagy in the maintenance of human T cell homeostasis. Liver-resident T cells with high basal autophagy were better able to maintain mitochondrial fitness. Our data suggest that non-resident T cells that infiltrate the liver are less-well adapted to the milieu and, without the requisite high basal autophagy levels, are more susceptible to mitochondrial depolarization. Similarly, we found that MAITs, another liver resident population (Salou et al., 2019) previously shown to maintain tight control of depolarized mitochondria (Zinser et al., 2018), are also characterized by high autophagy levels. Overall, these observations support the concept that tight mitochondrial quality control is essential for T cell homeostasis in the liver and that this is provided by enhanced autophagy levels. Future work is needed to investigate whether the depolarized mitochondria characteristic of intrahepatic T cells are accompanied by a high burden of cytosolic or mitochondrial ROS and whether ROS scavengers can abrogate their dependence on autophagy (Fisicaro et al., 2017, Pilipow et al., 2015). Alternatively, the requirement for mitophagy to replace damaged mitochondria may provide a ROS-independent mechanism by which autophagy adapts T cells to tissue residence.

A loss of control of mitochondrial fitness is a characteristic of exhausted T cells (Bengsch et al., 2016), in particular, HBV-specific T cells (Schurich et al., 2016). Exhausted HBV-specific T cells could be partially rejuvenated *in vitro* by correcting this mitochondrial defect with mitochondria-specific ROS scavengers (Fisicaro et al., 2017). Using MHC class I multimers, we were able to show that HBV-specific T cells circulating in chronically infected patients, which would have been primed and/or exposed to cognate antigens in the liver, had a higher level of autophagy when assayed in the blood relative to total CD8^+^ T cells or CMV-specific T cells. This suggested that virus-specific T cells can have enhanced autophagy levels imprinted on them by the environment in which they encounter antigens.

We investigated the autophagy-inducing properties of the prototypic liver cytokine IL-15 (Golden-Mason et al., 2004, Jiao et al., 2016), which can be produced by HSCs and hepatic macrophages (Golden-Mason et al., 2004, Winau et al., 2007, Zhou et al., 2018). IL-15 has been linked to the maintenance and imprinting of the T_RM_ cell phenotype (Holz et al., 2018, Mackay et al., 2015) and to autophagy induction in NK and NKT cells (Zhu et al., 2018). In this study, we show that IL-15 can directly induce autophagy in human T cells and that T_RM_ cell induction *in vitro* is dependent on autophagy. IL-15 has also been used for optimal expansion of antigen-specific T cells *in vivo* in melanoma models (Zeng et al., 2005), for stem-cell-like chimeric antigen receptor T (Tscm) cells (Hurton et al., 2016), and is crucial for persistence of inflationary MCMV-specific T cells (Baumann et al., 2018). We have previously shown that IL-15, in combination with IFN-α, can restore T cell effector function and proliferative capacity in exhausted HBV-specific T cells *in vivo* for mice and *in vitro* for humans (Di Scala et al., 2016). Whether autophagy is involved in shaping T cell differentiation and functionality in these settings should be investigated. Taken together, our data suggest that IL-15 or other means of inducing T cell autophagy may constitute a useful strategy to promote highly functional T cells with the capacity to reside in the liver and resist mitochondrial depolarization, for example, in the optimization of adoptive T cell therapy for hepatocellular carcinoma (HCC) (Qasim et al., 2015).

In summary, upregulation of autophagy in human liver-resident CD8^+^ memory T cells drives the acquisition of tissue-retention markers and protects against mitochondrial depolarization to optimize effector function. We demonstrate the capacity of the prototypic hepatic cytokine IL-15 to induce autophagy and suggest this imposes a cell-intrinsic adaptation to the liver niche on tissue-resident memory T cells.

## STAR★Methods

### Key Resources Table

**Table:** 

REAGENT or RESOURCE	SOURCE	IDENTIFIER
**Antibodies**		

APC anti-human sequestosome (SQSTM1; p62)	Abcam	Cat# Ab194721; RRID:AB_2728795
PE anti-human ⍺-smooth muscle actin ⍺-SMA clone 1A4	R and D Systems	Cat# IC1420P; RRID:AB_2223026
APC anti-human Intercellular Adhesion Molecule 1 (ICAM-1) clone HA58	eBioscience	Cat# 17-0549-41; RRID:AB_10718240
BUV805 anti-human CD45 clone HI30	BD biosciences	Cat# 564914; RRID:AB_2744401
BV711 anti-human CD3 clone OKT3	BioLegend	Cat# 317328; RRID:AB_2562907
BUV395 anti-human CD3 clone UCHT1	BD biosciences	Cat# 563546; RRID:AB_2744387
BV605 anti-human CD3 clone OKT3	BioLegend	Cat# 317322; RRID:AB_2561911
BUV395 anti-human CD4 clone SK3	BD biosciences	Cat# 563550; RRID:AB_2738273
APC-Cy7 anti-human CD4 clone RPA-T4	BioLegend	Cat# 300518; RRID:AB_314086
Pe-Cy7 anti-human CD4 clone OKT4	BioLegend	Cat# 317414; RRID:AB_571959
PerCP anti-human CD4 clone RMA4-5	BioLegend	Cat# 100538; RRID:AB_893325
Alexa Fluor700 anti-human CD8⍺ clone RPA-T8	BioLegend	Cat# 301028; RRID:AB_493745
PerCp-Cy5.5 anti-human CD8⍺ clone RPA-T8	BioLegend	Cat# 301032; RRID:AB_893422
BV786 anti-human CD8⍺ clone RPA-T8	BioLegend	Cat# 301046; RRID:AB_2563264
PE anti-human CD161 clone 191B8	Miltenyi Biotec	Cat# 130-092-677; RRID:AB_871632
PerCP-Cy5.5 anti-human pan ⍺β-TCR clone IP26	BioLegend	Cat# 306724; RRID:AB_2563002
APC anti-human pan ɣδ-TCR clone B3	BioLegend	Cat# 331212; RRID:AB_1089214
BV785 anti-human Va7.2 TCR clone 3C10	BioLegend	Cat# 351722, RRID:AB_2566042
BV510 anti-human CD19 clone SJ25C1	BD biosciences	Cat# 562947; RRID:AB_2737912
Pe-Cy7 anti-human CD56 clone NCAM16.2	BD biosciences	Cat# 335791; RRID:AB_399970
BV421 anti-human CD28 clone CD28.2	BioLegend	Cat# 302929; RRID:AB_2561341
Pe/Dazzle594 anti-human CD69 clone FN50	BioLegend	Cat# 310942; RRID:AB_2564277
BV605 anti-human CD69 clone FN50	BioLegend	Cat# 310938, RRID:AB_2562307
BV711 anti-human CD103 clone Ber-ACT8	BioLegend	Cat# 350222; RRID:AB_2629651
BV605 anti-human CD103 clone Ber-ACT8	BioLegend	Cat# 350218; RRID:AB_2564283
BV421 anti-human CXCR6 (CD186) clone K041E5	BioLegend	Cat# 356014; RRID:AB_2563873
APC anti-human CXCR6 (CD186) clone K041E5	BioLegend	Cat# 356006; RRID:AB_2562223
Pe-Cy7 anti-human CCR7 (CD197) clone 3D12	BD biosciences	Cat# 557648; RRID:AB_396765
Alexa Fluor700 anti-human CD45RA clone HI100	BD biosciences	Cat# 560673; RRID:AB_1727496
V450 anti-human Interferon-gamma (IFNɣ) clone B27	BD biosciences	Cat# 560371; RRID:AB_1645594
Alexa Fluor700 anti-human Granzyme B clone GB11	BD biosciences	Cat# 560213; RRID:AB_1645453
BV510 anti-human Perforin clone dG9	BioLegend	Cat# 308120; RRID:AB_2563829
PE-Cy7 anti-human Ki67 clone 20Raj1	eBioscience	Cat# 25-5699-42; RRID:AB_2573462
BV605 anti-human CD38 clone HIT2	BioLegend	Cat# 303532; RRID:AB_2562915
BV510 anti-human CD127 (IL7Rα) clone A019D5	BioLegend	Cat# 351331; RRID:AB_2561935
PE anti-human PD-1 (CD279) clone EH12.2h7	BioLegend	Cat# 329906; RRID:AB_940483
anti-human CD28 unconjugated clone CD28.2	Thermo Fisher Scientific	Cat# 16-0289-85; RRID:AB_468927
anti-human CD23 unconjugated clone OKT3	Thermo Fisher Scientific	Cat# 16-0037-85, RRID:AB_468855
Anti-human IL15 (blocking Antibody)	R&D systems	Cat# MAB247; RRID:AB_212457

**Biological Samples**		

Healthy human peripheral blood mononuclear cells	This paper	N/A
Intrahepatic lymphocytes isolated from perfusion fluid and explanted human liver tissue	This paper	N/A
Primary human hepatic stellate cells (HSCs)	This paper	N/A

**Chemicals, Peptides, and Recombinant Proteins**		

Ficoll-Paque PLUS density gradient media	GE Healthcare	Cat# 17144003
Pancoll Lymphocyte Separating Medium, human	Pan Biotech	Cat# P04-60125
Percoll density gradient media	GE Healthcare	Cat# 17089101
RPMI1640	Thermo Fisher Scientific	Cat# 21875
Collagenase IV	Thermo Fisher Scientific	Cat# 17104-019
DNase I	Roche	Cat# 11284932001
FBS	Sigma-Aldrich	Cat# F7524
Penicillin-streptomycin	Thermo Fisher Scientific	Cat# 15140122
Phosphate buffered saline	Thermo Fisher Scientific	Cat# 14190
Fixable live/dead Near-Infrared	Thermo Fisher Scientific	Cat# L10119
Fixable live/dead Violet	Thermo Fisher Scientific	Cat# L34955
Fixable live/dead Blue	Thermo Fisher Scientific	Cat# L23105
Brilliant Stain buffer	BD biosciences	Cat# 563794
Formaldehyde solution	Sigma-Aldrich	Cat# F8775
10X perm buffer	eBioscience	Cat# 00-5523-00
Brefeldin A	Sigma-Aldrich	Cat# B6542
Foxp3 / Transcription Factor staining buffer kit	eBioscience	Cat# 00-5523-00
ArC amine reactive compensation bead kit	Thermo Fisher Scientific	Cat# A10346
Bafilomycin A1	Sigma-Aldrich	Cat# B1793
Anti-mouse Ig compensation particle set	BD biosciences	Cat# 552843
RPMI1640 phenol-red free	Thermo Fisher Scientific	Cat# 11853-063
MRT68921 dihydrochloride	Sigma-Aldrich	Cat# SML1644 Petherick et al., 2015
3-Methyladenine	Sigma-Aldrich	Cat# M9281 Wu et al., 2010
MitoTEMPO	Sigma-Aldrich	Cat# SML0737 Fisicaro et al., 2017
N-acetyl-L-cysteine	Sigma-Aldrich	Cat# A9165
MitoTracker Deep Red FM	Thermo Fisher Scientific	Cat# M22426
MitoTracker Green FM	Thermo Fisher Scientific	Cat# M7514
Dimethyl sulfoxide	Sigma-Aldrich	Cat# D4540
Verapamil hydrochloride	Sigma-Aldrich	Cat# V4629
Cyclosporin A	Sigma-Aldrich	Cat# 30024
CellTrace violet	Thermo Fisher Scientific	Cat# C34557
MHC class I dextramers	Immudex	-
recombinant human IL-2	Peprotech	Cat# 200-02
recombinant human IL-15	R and D Systems	Cat# 247-ILB-005/CF
recombinant human TGFβ	BioLegend	Cat# 580702
Optiprep Density Gradient Medium	Sigma-Aldrich	Cat# D1556
Stellate Cell Medium	ScienCell Research Laboratories	Cat# 5301
Trypsin-EDTA (0.05%) phenol red	Thermo Fisher Scientific	Cat# 25300054
Recombinant human IL15-Rα-FC chimera protein	R and D Systems	Cat# 147-IR-100

**Critical Commercial Assays**		

FlowCellect Autophagy LC3 Antibody-based Assay Kit (LC3 clone 4E12)	Merck-millipore (now Luminex)	Cat# FCCH100171 Eng et al., 2010
Cyto-ID	Enzo LifeSciences	Cat# ENZ-51031-0050
panT cell isolation kit, human	Miltenyi Biotec	130-096-535

**Software and Algorithms**		

Prism version 7.0e	GraphPad	RRID:SCR_002798; https://www.graphpad.com/
FlowJo version 10.4.1 for mac	Tree Star	RRID:SCR_008520; https://www.flowjo.com
INSPIRE and IDEAS software	Merck-millipore (now Luminex)	-
R version 3.2.4	www.r-project.org	RRID:SCR_001905; http://www.rproject.org/

**Other**		

GentleMACS dissociator	Miltenyi Biotec	Cat# 130-093-235
LSRII Fortessa X20 cell analyzer	BD biosciences	-
LSRII cell analyzer	BD biosciences	-
Amnis ImageStream^X^ imaging flow cytometer	Merck-millipore (now Luminex)	-

### Lead Contact and Materials Availability

Further information and requests for resources and reagents should be directed to and will be fulfilled by the Lead Contact, Leo Swadling (l.swadling@ucl.ac.uk). This study did not generate any new unique reagents.

### Experimental Model and Subject Details

#### Subjects and recruitment

This study was approved by the local ethical boards of London-Brent (Research Ethics Committee reference number 16/LO/1699) and Brighton and Sussex (Research Ethics Committee reference number 11/LO/0421). Each participant gave written informed consent before inclusion. All storage of samples obtained complied with the requirement of the Data Protection Act 1998 and the Human Tissue Act 2004, issued by the UK parliament. The influence of gender of human subjects used in this study was only considered for data in Figure 4G, where significant inter-individual variation was observed. No influence of gender was seen (Mann-Whitney t test female versus male p = 0.57 not significant). The gender of participants was not routinely collected with anonymised clinical data for healthy volunteers and tissue donors.

#### Sample collection

Resected liver tissue from the healthy margins of tumor resections (colorectal metastases, adenocarcinoma, cholangiocarcinoma, hepatocellular carcinoma) and paired blood samples were obtained through the Tissue Access for Patient Benefit (TAPb) scheme at The Royal Free Hospital (approved by the University College London–Royal Free Hospital BioBank Ethical Review Committee; Research Ethics Committee reference number 11/WA/0077). Perfusion liquid was obtained from healthy livers prior to solid-organ transplantation (Research Ethics Committee reference 11/H0720/4).

For comparison, peripheral blood samples from healthy control individuals were included within the study (approved by the South East Coast Research Ethics Committee; Research Ethics Committee reference number 11/LO/0421; IRAS project number, 43993). All healthy control participants used within the study were anti-HBV, anti-hepatitis C, and anti-HIV antibody negative. Sample sizes are given in the legends for all experiments where individual data points are not shown.

### Method Details

#### PBMC and IHL isolation

PBMC were isolated by density centrifugation. Heparinised blood was layered on Ficoll-Hypaque Plus (GE Healthcare) or Pancoll (Pan Biotech) and was centrifuge for 20 minutes (mins) 800 g with slow acceleration and without brake. IHL were isolated from perfusion liquid by first concentrating the cells by centrifugation (300 g 10 mins) and resuspension in RPMI 1640 (Thermo Fisher Scientific). The concentrated cell suspension was then layered on Ficoll-Hypaque Plus as above.

Explanted liver tissue sections were cut into small pieces using scissors and were incubated for 30 min in 0.01% collagenase IV (Thermo Fisher Scientific) and 0.001% DNase I (Roche) at 37°C in a humidified atmosphere with 5% CO_2_. After enzymatic digestion, mechanical digestion was performed using a GentleMACS (Miltenyi Biotec). After full digestion, debris was removed by passing single cell suspension through 70 μM cell strainers (BD biosciences). Parenchymal cells were removed by centrifugation (400 g) on a 30% Percoll gradient (GE Healthcare). Finally, IHL were isolated by density centrifugation using Ficoll-Hypaque Plus as above.

All experiments using paired IHL and PBMC were performed *ex vivo* on freshly isolated cells. For ImageStream experiments frozen IHL isolated from perfusion fluid were used. All experiments using PBMC from healthy controls used frozen PBMC. PBMC and IHL were frozen in 10% DMSO (Sigma-Aldrich) in Isopropanol containers (−1°C/minute) at 5-20 × 10^6^ PBMC/ml in cryovials. Thawing was performed by gentle agitation at 37°C with rapid dilution in RPMI containing 10% fetal bovince serum (FBS; Sigma-Aldrich) and 0.001% DNase I. RPMI 1640 + 10% FBS, 100 U/ml penicillin/streptomycin was used for T cell culture media (R10).

#### Flow cytometry – Surface, intracellular, intranuclear, and cytokine staining

For multiparametric flow cytometry cells were plated in 96-well round-bottomed plates (200,000-1,000,000) and washed once in PBS (Phosphate buffered saline; Thermo Fisher Scientific) and stained with a fixable live/Dead dye (Thermo Fisher Scientific) for 20 mins at 4°C in PBS. Cells were washed again in PBS, and incubated with saturating concentrations of surface monoclonal antibodies (mAbs) diluted in 50% Brilliant violet buffer (BD biosciences) and 50% PBS for 30 min at 4°C unless stated. For surface marker assessment alone cells were then fixed with 1% formaldehyde (Sigma-Aldrich) in PBS for 20 mins 4°C and washed twice in PBS before being analyzed on a flow cytometer.

For intracellular and intranuclear staining, after surface Ab staining cells were resuspended in fix/perm buffer (Foxp3 / Transcription Factor staining buffer kit, fix perm concentrate diluted 1:3 in fix/perm diluent) for 45-60 mins at 4°C. Cells were then washed in 1x perm buffer (10x perm buffer Foxp3 / Transcription Factor staining buffer kit diluted to 1X in ddH_2_O) and saturating concentrations of antibodies against intranuclear targets were stained in 1X perm buffer for 30-45 mins 4°C. Cells were washed twice in PBS then analyzed by flow cytometry. For intracellular cytokine staining PBMC 1 hour after stimulation 1 μg/ml brefeldin A (Sigma-Aldrich) was added and PBMC were incubated for 16 hours at 37°C in a humidified atmosphere with 5% CO_2_. For stimulation by CD3-crosslinking with co-stimulation anti-CD3 and anti-CD28 (eBioscience) antibodies were diluted to 0.5 μg/ml in PBS and incubated on Nunclon delta surface 96-well round-bottom plates (Thermo Fisher Scientific) for 1 hour. Plates were washed three times with PBS and then cells were added in culture media.

All samples were acquired in PBS on LSRII or Fortessa X20 flow cytometers (BD biosciences) and analyzed using FlowJo (version 10.4.1 for mac, Tree Star). Single stain controls were prepared with cells where possible or anti-mouse IgG beads (BD biosciences) and Arc-Amine reactive beads (Thermo Fisher Scientific).

#### Flow cytometry based LC3-I quantification

To quantify the number of autophagosome *ex vivo*, or the accumulation of autophagosomes (after inhibiting autophagosome degradation) as proxies for the level of autophagy or autophagic flux respectively, the FlowCellect Autophagy LC3 Antibody-based Assay Kit (Merck Millipore, now Luminex) was used (Eng et al., 2010). A proprietary permeabilisation buffer (Autophagy reagent B) is used to selectively extract non-autophagosome associated cytosolic LC3-I; therefore, when the anti-LC3 antibody is added only the lipidated form LC3-II incorporated within autophagosomes remains and is stained. The fluorescence of the anti-LC3-FITC antibody can then be used to quantify autophagosomes.

For *ex vivo* assessment of autophagy levels autophagosomes were stained by flow cytometry using a fluorescently labeled anti-LC3 antibody (clone 4E12). PBMC or IHL were washed, stained for live/dead marker and then surface markers as above. Cells were then centrifuged at 300 g 4 mins 4°C and resuspended in 50 μL autophagy reagent B permeabilisation buffer (Diluted 1:10 in ddH20) per well and immediately centrifuged 300 g 4 mins 4°C and resuspended in anti-LC3-FITC (1:20 50ul/well) diluted in assay buffer (assay buffer concentrate is diluted 1:5 in ddH20) and incubated at 4°C for 30 mins. Cells were then washed in PBS, resuspended in 1% formaldehyde to fix for 20 mins at 4°C, washed in PBS, and run on a flow cytometer.

The geometric mean fluorescence intensity (geoMFI) of LC3 *ex vivo* without addition of bafilomycin A1 (“unblocked”) was used to define the basal autophagy level. To measure the accumulation of autophagosomes over time 0.1 μM bafilomycin A1 (“blocked”; Sigma-Aldrich; diluted in DMSO; (Klionsky et al., 2016, Clarke et al., 2018, Puleston et al., 2014), was added to cell cultures overnight (Figures 1, 2 [excluding 2F], 3, 5E, 5F, S1C, and S1D) for paired IHL and PBMC samples, or for 3 hours for all other experiments (Figures 2F, 4, and 5; Figures 5G and 5H are unblocked) (See Figures S1B, S1C, S2, S3C and S3E for comparisons of unblocked and bafilomycin A1 blocked staining). The ratio of geoMFI LC3 (flux) for blocked versus unblocked was calculated as follows: (+bafA1 blocked geoMFI LC3 - unblocked geoMFI LC3) / unblocked geoMFI LC3.

When LC3 was co-stained with intracellular or intranuclear markers (Figures 2C-2E and 4A–4D) PBMC were fixed with fix/perm buffer (Foxp3/Transcription Factor staining buffer kit) as above and anti-LC3 was added to the intracellular antibody cocktail in perm buffer (Foxp3 / Transcription Factor staining buffer kit). Non-autophagosome associated cytosolic LC3-I is not selectively extracted using reagent B in this protocol, therefore, a common background of LC3-I is also labeled. PBMC samples were stained in parallel using the FlowCellect Autophagy LC3 Antibody-based Assay Kit as above or the BD Foxp3/Transcription Factor staining buffer kit (Figure S3E and S3F). A strong positive correlation between the two staining conditions confirmed that the samples with higher rates of autophagy by the standard protocol also had a higher level of LC3 staining when stained with the intracellular protocol, and that the LC3-I background level was consistent across samples and treatments. Samples stained by the intracellular protocol or FlowCellect Kit were measured on separate Flow Cytometers with different panels however, so direct comparisons of the MFI magnitude cannot be made.

Intracellular staining of p62 (sequestosome 1; SQSTM1; Abcam) was performed as described in Clarke et al. 2015. IHL were incubated overnight with or without bafA1 (0.1 μM). The following day after fixable live/dead stain and surface Ab staining as above cells were fixed in 1% formaldehyde for 20 mins at 4°C. Cells were then permeabilised in 1x perm buffer (10x perm buffer Foxp3 / Transcription Factor staining buffer kit diluted to 1X in ddH_2_O) for 20 mins at 4°C and then saturating concentration of anti-p62-alexa647 was added in 1X perm buffer for 30 mins 4°C. Cells were washed in 1x perm buffer and then PBS before acquiring. The ratio of geoMFI p62 (sequestosome 1; SQSTM1; abcam) (flux) for blocked versus unblocked was calculated as follows: (+bafA1 blocked geoMFI p62 - unblocked geoMFI p62) / unblocked geoMFI p62.

#### Flow cytometry based autophagosome quantification using Cyto-ID

Cyto-ID is a proprietary dye that includes titratable moieties specific for staining autophagic vesicles (Enzo Lifesciences). To measure autophagy in T cells using the Cyto-ID autophagy detection kit (ENZ-51031-0050) PBMC and IHL were thawed (as above) and rested overnight in R10 culture media in 96-well plates at 0.5 × 10^6^ PBMC per well at 37°C in a humidified atmosphere with 5% CO_2_. Plates were centrifuged at 300 g 4 mins RT and cell pellets were resuspended in pre-warmed (37’C) RPMI without phenol red (Thermo Fisher Scientific) containing 5% FBS, 100 U/ml penicillin/streptomycin and Cyto-ID dye diluted 1:4000. Cells were incubated with Cyto-ID at 37°C in a humidified atmosphere with 5% CO_2_ for 30 mins before being washed with the Cyto-ID kit assay buffer diluted to 1X in sterile distilled H_2_O. Cells were then stained with fixable live/dead, stained with surface antibodies, fixed with 1% formaldehyde, and analyzed by flow cytometry as above for the surface staining protocol.

#### Autophagy inhibitors

MRT68921 dihydrochloride (Sigma-Aldrich; used at 1 or 10 μM for 6 day or overnight cultures respectively) is an autophagy specific ULK1 and ULK2 kinases inhibitor (Klionsky et al., 2016, Petherick et al., 2015). 3-Methyladenine (3-MA; Sigma-Aldrich; 0.5 mM) is a type I and III Phosphatidylinositol 3-kinases (PI-3K) inhibitor (Wu et al., 2010).

#### MitoTracker staining

Freshly isolated IHL and PBMC were stained *ex vivo* with cell permeable mitochondrial dyes by adding them directly to culture media. MitoTracker Deep Red FM and MitoTracker Green FM (Thermo Fisher Scientific) were added to a final concentration of 12.5 nM and 25 nM in the well respectively and were stained for 20 mins at 37°C in a humidified atmosphere with 5% CO_2_. Cells were then washed in PBS and stained with fixable live/dead and surface antibodies as above. Cells were left unfixed for flow cytometric analysis. For overnight inhibition of autophagy (Figures 4G) bafilomycin A1 (0.1 μM), Reagent A (Chloroquine diphosphate, FlowCellect Autophagy LC3 Antibody-based Assay Kit), MRT68921 dihydrochloride, or DMSO vehicle were added to R10 culture media and cells were stained for MitoTrackers as above after ∼16 hours culture.

For inhibition of efflux pumps verapamil (Sigma-Aldrich; 50 uM) and cyclosporin A (Sigma-Aldrich; 50 uM) were added directly to culture media with the mitoTracker dyes and incubated for 20 mins at 37°C in a humidified atmosphere with 5% CO_2_ and mitoTracker staining was as above.

#### ImageStream

ImageStream has been used extensively to directly image intracellular autophagosomes using fluorescently labeled anti-LC3 antibodies (Clarke et al., 2018, Klionsky et al., 2016, Phadwal et al., 2012, Sanderson and Simon, 2017, Puleston et al., 2014).

IHL and PBMC were thawed as above and were cultured overnight (16 hours) in R10 culture media supplemented with 0.1 μM bafilomycin A1. Cells were washed and T cells were isolated using negative magnetic bead selection (panT cell isolation kit, Miltenyi Biotec) according to the manufacturer’s instructions. T cells were collected on ice, counted, stained as above for surface markers and intracellular LC3 and fixed with 1% formaldehyde. The following panel was used: Camera 1; Ch1 Bright field, Ch2 LC3-FITC, Ch3 CD4-PE, Ch5 CD8-PerCp-Cy5.5, Ch6 SSC. Camera 2 Ch8 CD69-V500, Ch10 CD103-BV605, Ch12 Near Infrared-fixable live/dead.

Amnis ImageStream^X^ imaging flow cytometer (MERK-Millipore) fitted with a 60 × microscope objective was used for cell imaging. Raw image files were acquired using INSPIRE software. After acquisition, a compensation matrix was applied to the data to correct for spectral overlap. Data analysis was done using IDEAS software, displaying cells using gradient RMS for the bright field channel to exclude out-of-focus cells and a combined area to aspect ratio dot plot ensured gating on single-cell events (see Figure S3 for gating and staining controls).

#### T cell proliferation assay

Frozen PBMC were thawed at 37°C as described above and washed twice with sterile PBS. PBMC were resuspended in 1 mL R10 culture media (2-10 × 10^6^ PBMC) and 0.5 μL of 5 mM stock CellTrace violet (Thermo Fisher Scientific) was added per sample with mixing. PBMC were stained in the dark for 10 mins at 37°C in a humidified atmosphere with 5% CO_2_. Ten-times volume of cold R10 was added to stop the staining reaction, and cells were incubated for 5 mins on ice. Cells were washed in PBS and incubated for 5 mins at 37°C before being transferred to a new tube and were washed again in R10. CTV stained and unstained control PBMC were plated in 48-well plates (0.5 × 10^6^ PBMC in 0.5 mL R10) stimulated with plate bound anti-CD3/CD28 cross-linking antibodies (0.5 μg/ml each added for 1 hr in PBS, washed with 3 × 1 mL PBS) for 5 days, or in uncoated wells (unstimulated control). At the end of the 5 days, PBMC were harvested from the 48-well plate and were transferred to a 96-well plate and were incubated with bafilomycin A1 for 3 hours, before being washed and stained for fixable live/dead, surface Abs, and LC3 as above.

#### MHC class I dextramer staining for the identification of antigenspecific T cells

HBV-specific HLA-A^∗^02-restricted dextramers (Immudex) of the following specificities were used: core_18–27_ (FLPSDFFPSV), envelope_183–191_ (FLLTRILTI), envelope_335–342_ (WLS LLVPFV), envelope_338–347_ (LLVPFVQWFV), envelope_348–357_ (GLSPTVWLSV), polymerase_455–463_ (GLSRYVARL), and polymerase_502–510_ (KLHLYSHPI). CMV specific CD8^+^ T cell responses were tracked using an HLA-A^∗^02-restricted dextramers (Immudex) loaded with the pp65_495-504_ (NLVPMVATV) peptide. PBMC were thawed, rested for 1 hour, and then incubated at 37°C in a humidified atmosphere with 5% CO_2_ overnight with bafilomycin A1 (0.1 μM). PBMC were washed in PBS and were stained with dextramers at room temperature (20mins) in PBS, washed twice in PBS before further mAb staining as described above. During analysis, stringent gating criteria were applied with doublet and dead cell exclusion to minimize nonspecific binding contamination. A dextramer loaded with an irrelevant peptide was stained in parallel to assess non-specific binding. Dextramer clouds of less than 50 cells were excluded from analysis.

#### *In vitro* Induction of T_RM_ phenotype

For *in vitro* tissue-resident T cell induction, PBMC from healthy controls were incubated at 5 × 10^5^ cells/well in 0.5 mL R10 culture media (supplemented with 20 IU/ml recombinant human IL2 at day 0 and day 3, Miltenyi Biotec) for 6 days with different combinations of cytokines (recombinant human TGFβ, rhTGFβ, 50 ng/ml; recombinant human IL15, rhIL15, 50 ng/ml). On day 6 PBMC were harvested from the 48-well plate, transferred to a 96-well plate and were incubated with bafilomycin A1 for 3 hours, before being washed and stained for fixable live/dead, surface Abs, and LC3 as above. Autophagy inhibitors were added throughout the culture, at day 0, day 3, and during the 3 hour incubation with bafilomycin A1.

For 3 day cultures, PBMC were plated in R10 culture media supplemented with different doses of IL15, without IL2. On day 3 PBMC were harvested from the 48-well plate, transferred to a 96-well plate and were incubated with bafilomycin A1 for 3 hours, before being washed and stained for fixable live/dead, surface Abs, and LC3 as above.

#### Primary human hepatic stellate cell co-culture

For extraction of primary human hepatic stellate cells (HSCs), fresh post-resection liver tissue was washed thoroughly, passed through a tissue press and digested with DNase I (0.001%) and collagenase IV (0.01%). Cellular homogenate was filtered through a 70 μm cell strainer and centrifuged at low speed to remove remaining parenchymal cells, then at 450 g to wash the cells. The remaining cells were layered for density gradient isolation using Optiprep (Sigma-Aldrich). After isolation, HSCs were suspended in Stellate Cell Medium (ScienCell Research Laboratories), plated at a density of 5 × 10^4^ cells/cm in tissue culture flasks and cultured at 37°C in a humidified atmosphere with 5% CO_2_. On day 2, 3 or 4, cell debris and non-adherent cells were removed by washing. When cultures reached confluence, cells were trypsinised and re-plated; cells were passaged twice before freezing.

Pre-isolated HSCs were thawed and cultured in 25 cm^2^ tissue culture flasks in Stellate Cell Medium to approximately 90% confluence. Cells were detached with trypsin–EDTA (Thermo Fisher Scientific), re-plated in 48-well plates in Stellate Cell Media (5,000-20,000 HSCs per well) and left for 36-48 hours to adhere. Media was removed and 5 × 10^5^ PBMC/well were added with R10 culture media and cells were co-cultured for 3 days. rhIL15Rα-Fc chimera (0.01 μg/ml; R&D systems) was added to R10 culture media with PBMC prior to being plated on HSCs. On day 3 PBMC were harvested from the 48-well plate, transferred to a 96-well plate and were washed and stained for fixable live/dead, surface Abs, and LC3 as above. To confirm the differentiation status of HSCs a sample was surface stained with ⍺-SMA and ICAM-1 at the end of the 3 days culture.

### Quantification and Statistical Analysis

#### t-Distributed Stochastic Neighbor Embedding (tSNE) analysis

The dimension reduction algorithm tSNE was applied to concatenated flow cytometry data (∼ 180,000 cells) from 2 paired IHL and PBMC samples using default parameters (iterations, 1,000; perplexity, 20; and θ, 0.5) in FlowJo. tSNE was applied to expression data for CD3, CD4, CD8⍺, CD19, CD103, CD69, pan-ɣδ T cell Receptor (TCR), pan-⍺β TCR, CD161, CD56, and LC3 after pre-gating on single, live, CD45^+^ lymphocytes.

#### Statistical analysis

Statistical analyses were performed in either Prism (Graph-Pad Software, version 7.0e) or R version 3.2.4 using appropriate methods as indicated in the legends (Mann-Whitney t test; Wilcoxon Signed-rank t test; Kruskal-Wallis test [ANOVA] for unpaired non-parametric multiple comparisons, Friedman test (ANOVA) for pairwise non-parametric multiple comparisons, both with Dunn’s post hoc test; Spearman’s Rank Order Correlation) with significant differences marked on all figures. All tests were performed as two-tailed tests, and for all tests, significance levels were defined as ^∗^, p < 0.05; ^∗∗^, p < 0.01; ^∗∗∗^, p < 0.001; and ^∗∗∗∗^, p < 0.0001.

### Data and Code availability

This study did not generate any unique datasets or code.

## Supplemental Information

Document S1. Figures S1–S5 and Table S1

Document S2. Article plus Supplemental Information
