# Supplementary material for: Human Liver Memory CD8+ T Cells Use Autophagy for Tissue Residence
Source: Cell Rep. 2020 Jan 21;30(3):687–698.e6. doi: 10.1016/j.celrep.2019.12.050 (PMC6988113; doi:10.1016/j.celrep.2019.12.050)
Supplement: Document S1. Figures S1–S5 and Table S1 [file mmc1.pdf]

Cell Reports, Volume 30

Supplemental Information

Human Liver Memory $CD8^+$ T Cells Use Autophagy for Tissue Residence

Leo Swadling, Laura J. Pallett, Mariana O. Diniz, Josephine M. Baker, Oliver E. Amin, Kerstin A. Stegmann, Alice R. Burton, Nathalie M. Schmidt, Anna Jeffery-Smith, Nekisa Zakeri, Kornelija Suveizdyte, Farid Froghi, Giuseppe Fusai, William M. Rosenberg, Brian R. Davidson, Anna Schurich, A. Katharina Simon, and Mala K. Maini

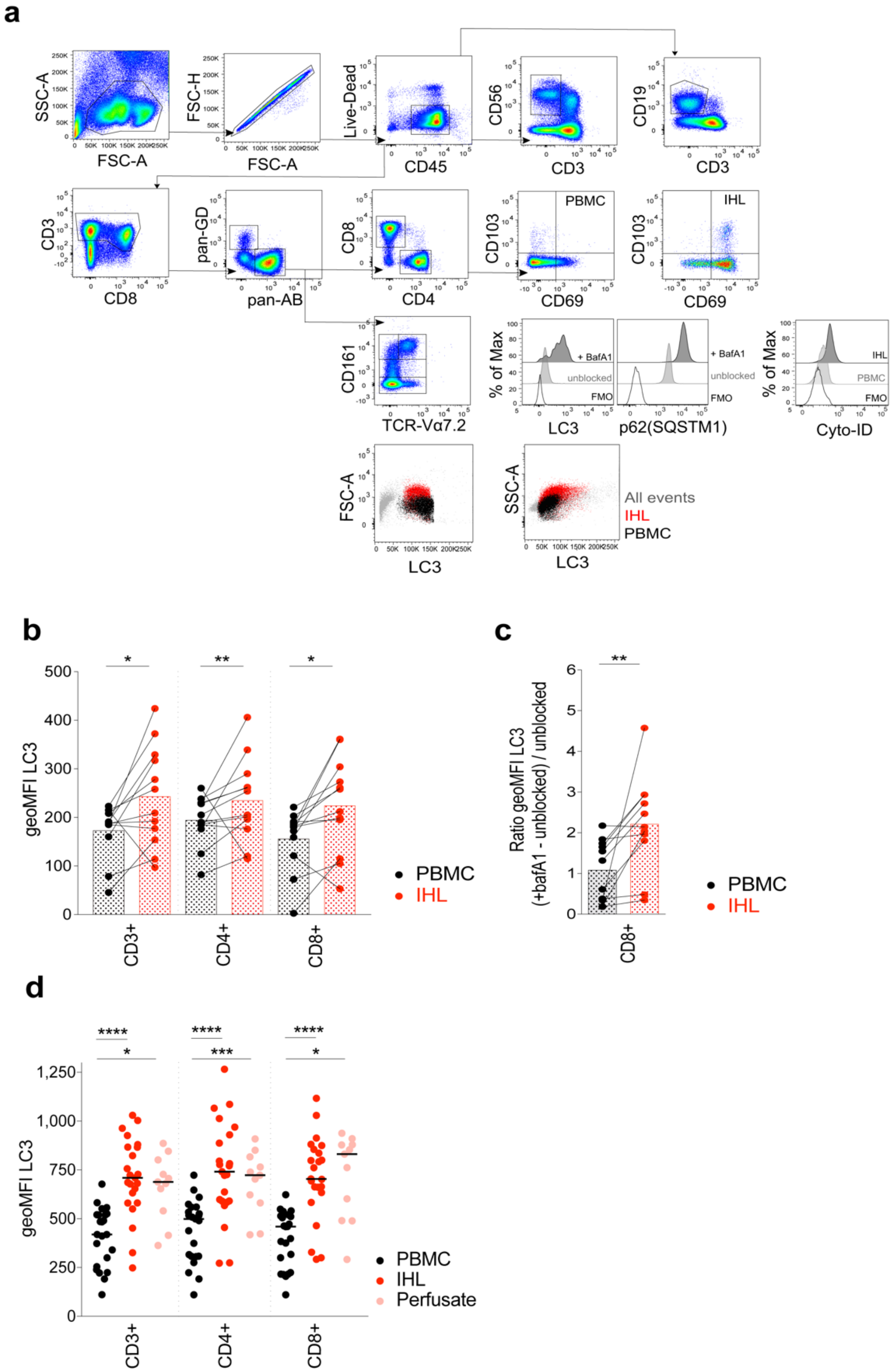

Figure S1. Sequential gating strategy for lymphocyte subsets and LC3 liver staining controls; Related to Figure 1; (a) Sequential gating strategy to identify: lymphocytes (SSC-A vs. FSC-

A)/single cells (FSC-H vs. FSC-A)/live CD45$^+$ lymphocytes (Fixable live/dead$^-$ CD45$^+$)/NK cells (CD56$^+$ CD3$^-$), B cells (CD19$^+$ CD3$^-$), T cells (CD3$^+$)/pan-γδ and pan-αβ T cells (pan-γδ TCR vs. pan-αβ TCR)/MAITs (CD161^{hi} TCR-Vα 7.2$^+$) and CD161mids (CD161^{mid} TCR-Vα 7.2$^-$), CD4+ and CD8$^+$ T cells (CD4 vs. CD8)/tissue-resident T cells (CD103 vs. CD69; shown for PBMC and IHL). Also shown are example histograms of LC3 and p62 (Sequestosome-1, SQSTM1) staining of IHL treated with bafilomycin A1 (bafA1) overnight or untreated (DMSO vehicle), and Cyto-ID staining of IHL and PBMC, all gated on CD8+ T cells with example fluorescence minus one stains (FMO). FSC-A vs. LC3 and SSC-A vs. LC3 plots are shown for all events (grey), CD3$^+$ T cells in PBMC (black) and CD3$^+$ T cells in IHL (red), showing a higher LC3 staining for cells of equivalent size and granularity in IHL samples relative to PBMC samples. **(b)** Summary data of the geoMFI of LC3 on CD3$^+$, CD4$^+$, and CD8$^+$ T cells in paired PBMC and IHL samples *ex vivo* without bafA1 treatment (unblocked; bars at mean; 12 biological replicates). **(c)** The ratio of geoMFI LC3 between accumulated (blocked; + bafA1) and basal (unblocked) samples for CD8$^+$ T cells from paired PBMC and IHL samples calculated as follows: (geoMFI LC3 with bafA1– unblocked geoMFI LC3) / unblocked geoMFI LC3; 11 biological replicates). (**d**) Summary data for CD3$^+$, CD4$^+$, and CD8$^+$ T cells in perfusion fluid (perfusate) and paired PBMC and IHL samples *ex vivo.* bafA1 treated (23 biological replicates for paired blood and IHL, 11 biological replicates for perfusates). (**b, c**) Wilcoxon paired t-tests. (**d**) Kruskal-Wallis (ANOVA) with Dunn's post hoc test for multiple unpaired comparisons. $^*P < 0.05$; $^{**}P < 0.005$; $^{***}P < 0.001$; $^{****}P < 0.0001$.

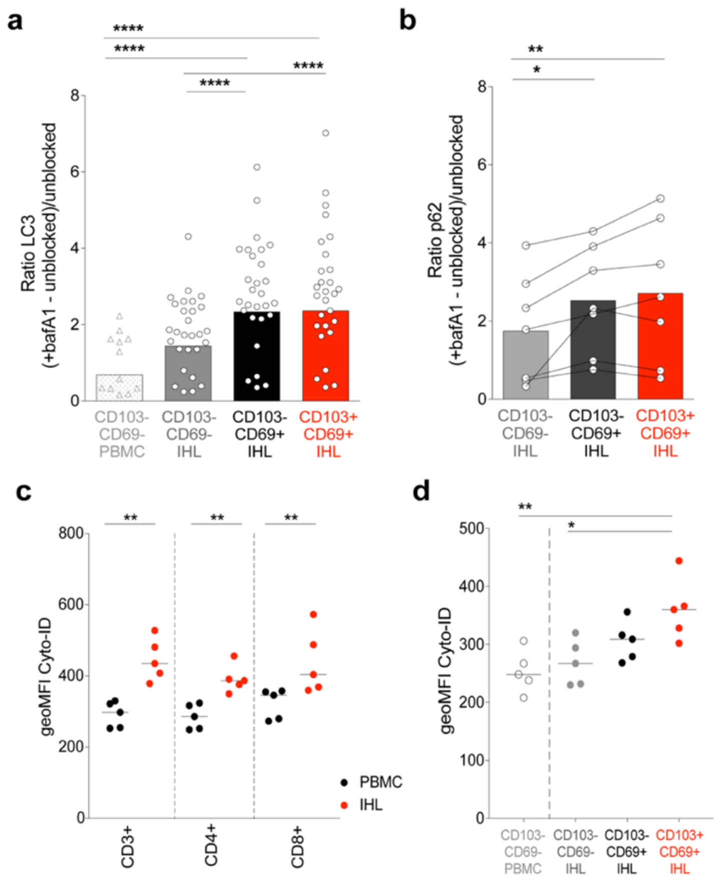

Figure S2. Autophagic flux is highest in CD8^{+} T$_{RM}$ in the liver when normalising for basal autophagy levels; Related to Figure 3; (a) The ratio of the geoMFI of LC3 (12 biological replicates PBMC, 21 biological replicates IHL) or **(b)** p62 (Sequestosome-1, SQSTM1; 7 biological replicates) between blocked (+bafA1) and unblocked samples for liver-resident (CD69^{+}CD103^{-} [black] and CD69^{+}CD103^{+} [red] subsets) and non-resident liver infiltrating T cells (CD69^{-}CD103^{-} [light grey]) in the human liver and CD69^{-}CD103^{-} CD8^{+} T cells in paired PBMC (LC3 only). The ratio was calculated as follows: (+bafA1 blocked geoMFI marker - unblocked geoMFI marker) / unblocked geoMFI marker. **(c-d)** Summary data for the geoMFI of Cyto-ID on **(c)** CD3^{+}, CD4^{+}, and CD8^{+} T cells in thawed PBMC and IHL samples and **(d)** on liver-resident (CD69^{+}CD103^{-} [black] and CD69^{+}CD103^{+} [red] subsets) and non-resident liver infiltrating T cells (CD69^{-}CD103^{-} [grey]) from IHL or PBMC (white). Bars at geometric median (example histograms **Figure S1a**; 5 biological replicates). **(a, d)** Kruskal-Wallis (ANOVA) with Dunn's post hoc test for multiple unpaired comparisons (PBMC vs. IHL). **(a, b)** Friedman test (ANOVA) with Dunn's post hoc test multiple for paired comparisons (between IHL subsets). **(c)** Mann-Whitney unpaired t-test for unpaired PBMC vs. IHL. *$P < 0.05$; **$P < 0.005$; ****$P < 0.0001$.

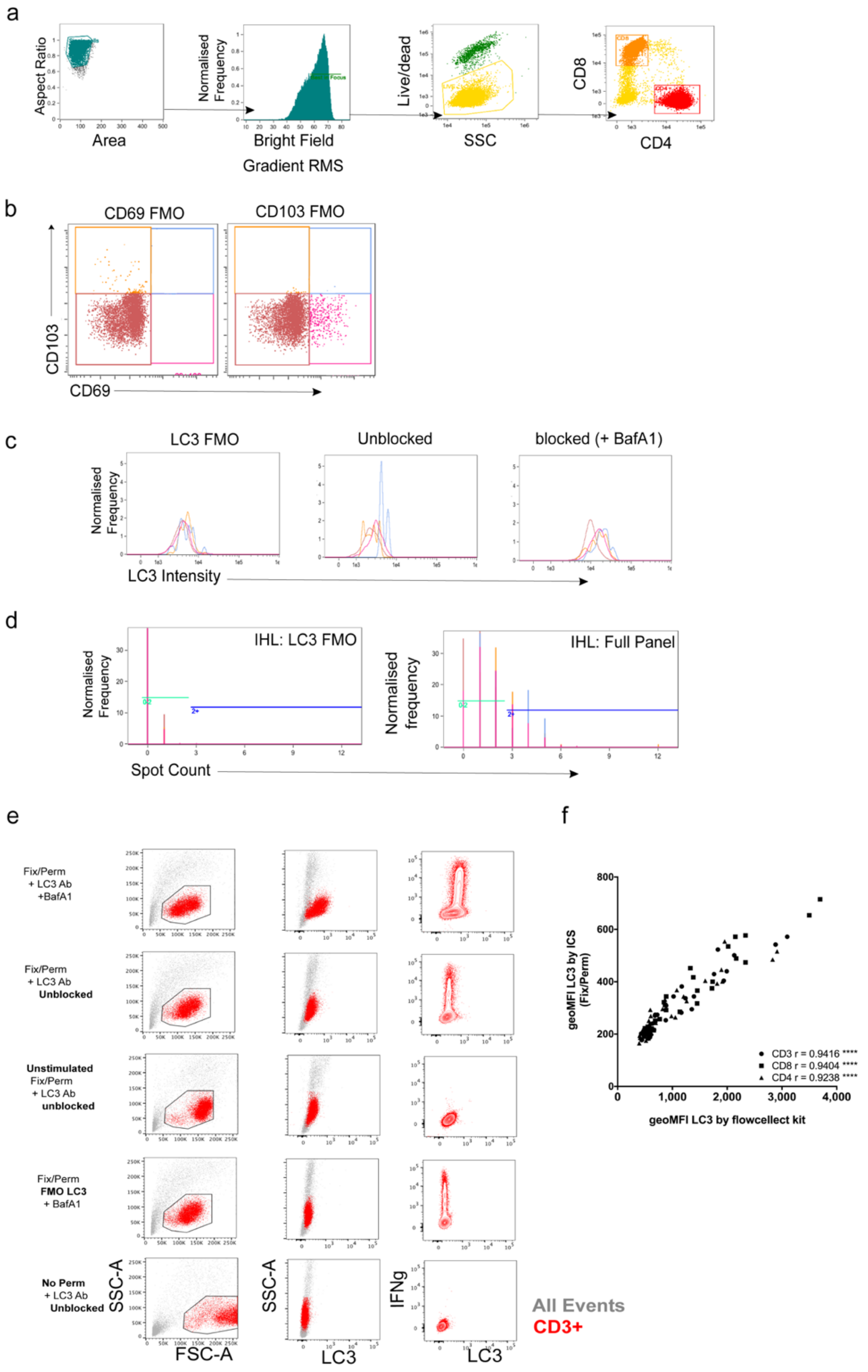

Figure S3. Staining Controls for ImageStream and Intracellular Cytokine staining; Related to Figures 3 and 4; (a) Sequential gating strategy for ImageStream data to identify: single cells

(Aspect ratio vs. Area)/ cells in focus (histogram of brightfield gradient RMS)/ live cells (fixable live/dead vs. SSC)/ $CD8^+$ and $CD4^+$ T cells (CD8 vs. CD4) and **(b)** tissue-resident subsets; $CD69^-CD103^-$ (red), $CD69^+CD103^-$ (pink), $CD69^-CD103^+$ (orange), $CD69^+CD103^+$ (blue; DP). **(c)** Histograms of LC3 intensity for LC3 fluorescence minus one (FMO), unblocked (no bafilomycin A1; bafA1) and blocked (+ bafA1) showing traces for each T_{RM} subset as shown in **(b;** PBMC gated on $CD8^+$). **(d)** Stacked histograms of LC3 puncta counts showing LC3 FMO (FMO) control or LC3 stained IHL (both overnight bafA1) coloured by T_{RM} subset as in **(b)**. **(e)** Healthy human PBMC were stimulated overnight with anti-CD3/CD28 in the presence of brefeldin A. Autophagosomal degradation was blocked with bafA1 for the final 3 hours of culture. Cells were fixed (Fix/perm buffer, eBioscience TF staining kit), permeabilised (diluted 10X perm buffer, eBioscience TF staining kit), and stained with anti-human LC3 antibody (FlowCellect kit) and anti-human IFNγ antibody (Top row). Controls were run as above but with the following changes: unblocked (no bafA1; second row); unstimulated and unblocked (no anti-CD3/CD28) to show the lack of IFNγ staining when unstimulated (third row); unstained for anti-LC3 to show a FMO control for the FITC-labelled anti-LC3 antibody (Fourth row); unpermeabilised and unblocked to show a loss of IFNγ and LC3 staining when cells are not permeabilised (Fifth row). Plots show SSC-A/FSC-A and SSC-A/LC3 gated on all events or $CD3^+$ (red) and IFNγ /LC3 for $CD3^+$ T cells. **(f)** Healthy human PBMC were stimulated overnight with anti-CD3/CD28 and stained as above for intracellular cytokine staining (ICS) or they were stained according to the FlowCellect LC3 staining kit in parallel (gated on lymphocytes/singlets/live/$CD45^+$/pan-αβ TCR^+/$CD3^+$, $CD4^+$ or $CD8^{+;}$ 31 biological replicates). Spearman's rank correlation. ****$P < 0.0001$.

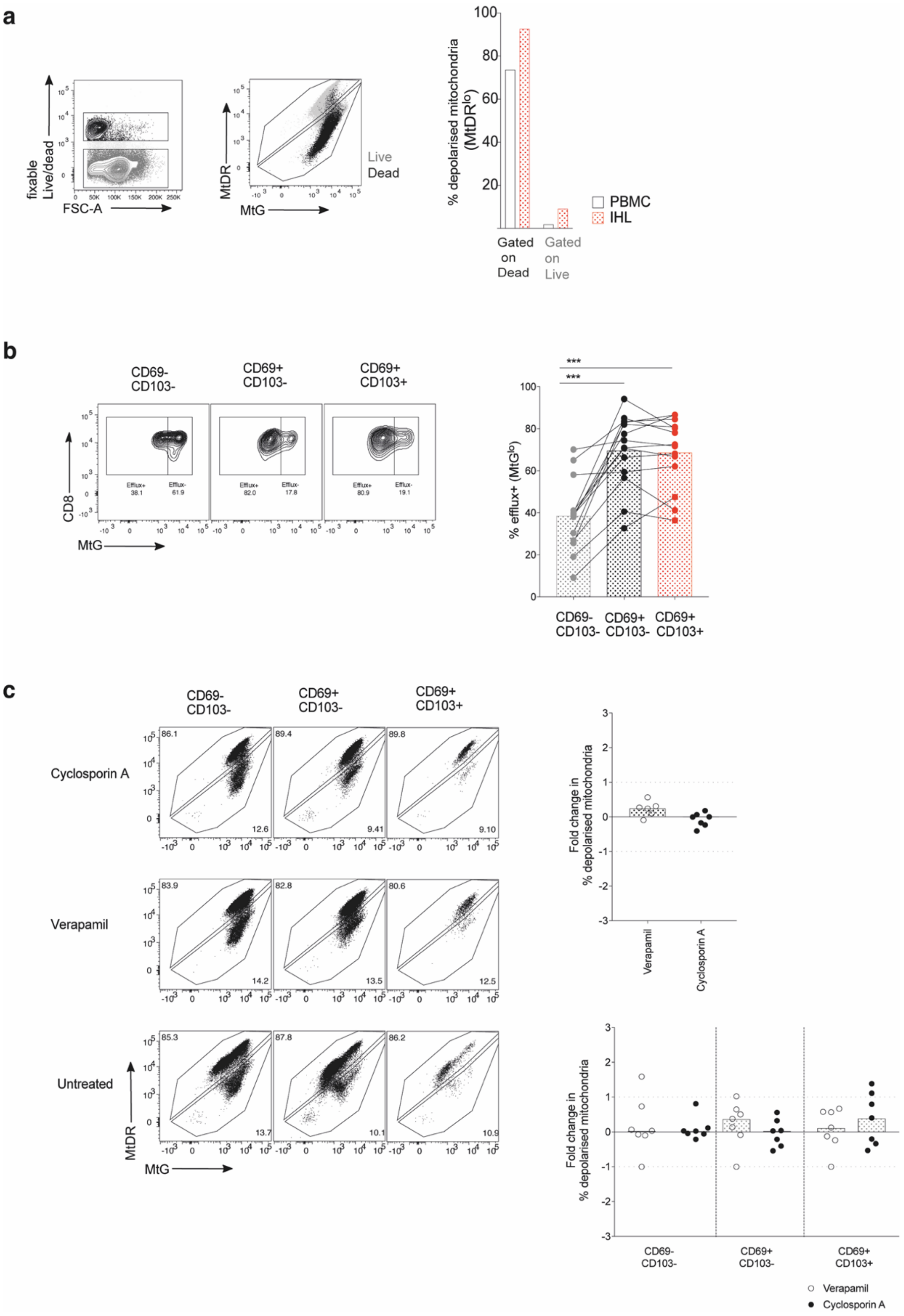

Figure S4. Related to Figure 4e-g; Mitochondrial staining of human T cells, staining controls; **(a)** Example plots of fixable live/dead stain (gated on total lymphocytes) and mitoTracker deep

red (MtDR) vs. mitoTracker green (MtG) co-staining of total live (fixable live/dead-; grey) and dead (fixable live/dead+; black) peripheral CD3+ T cells, and summary data for percentage of depolarised mitochondria ($MtDR^{lo}$) within live or dead gate for *ex vivo* stained PBMC and IHL (bars at median, 7 biological replicates). **(b)** Example plots of $efflux^{-}$ (MtG^{hi}) and $efflux^{+}$ (MtG^{lo}) $CD8^{+}$ T cells and summary data for the percentage of efflux+ T_{RM} in *ex vivo* stained IHL (bars at mean; 14 biological replicates). **(c)** Example plots and summary data for percentage of total $CD8^{+}$ T cells (top) or $CD8^{+}$ T_{RM} subsets (bottom) with depolarised mitochondria ($MtDR^{lo}$) *ex vivo* within IHL samples after treatment with cyclosporin A (50 μM) or verapamil (50 μM; fold change calculated as (treated – untreated)/untreated); 6-7 biological replicates). **(b)** Friedman test (ANOVA) with Dunn's post hoc test multiple for paired comparisons (between IHL subsets). **(c)** Kruskal-Wallis (ANOVA) with Dunn's post hoc test for multiple unpaired comparisons (untreated vs. CSA vs. verapamil for total CD8+ T cells and for each T_{RM} subset). ***$P < 0.001$.

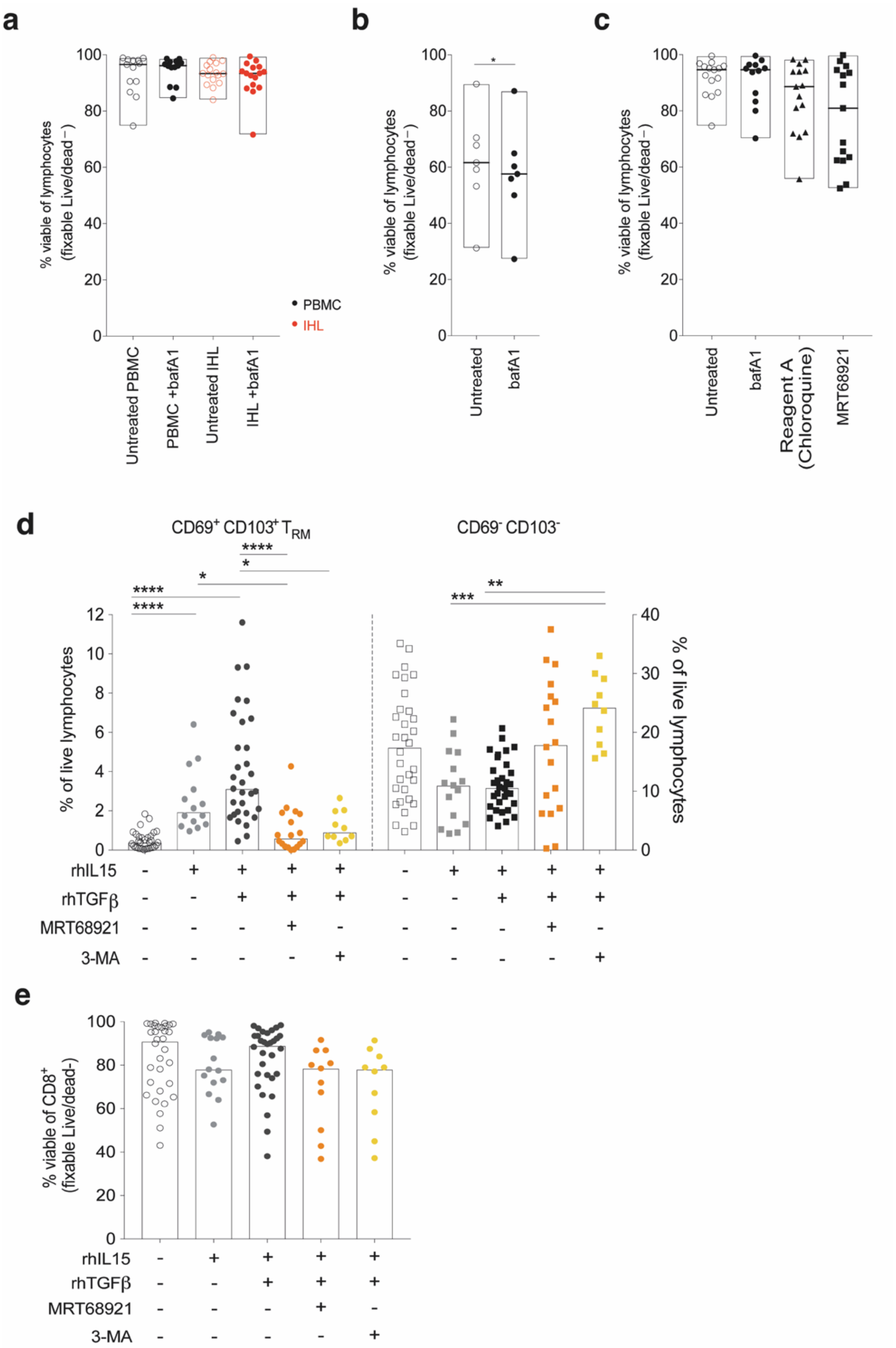

Figure S5. Lymphocyte viability after overnight or six day culture in the presence of autophagy inhibitors; Related to Figures S1c, S2b, 4g and 5e-f; (a) The percentage of fixable

live/dead$^-$ (viable) lymphocytes in PBMC and IHL samples after overnight culture with and without bafilomycinA1 (LC3-I staining in **Figure S1c;** 13-16 biological replicates). **(b)** The percentage of fixable live/dead$^-$ (viable) lymphocytes in IHL samples after overnight culture with and without bafilomycinA1 (p62 staining in **Figure S2b;** 7 biological replicates). **(c)** The percentage of fixable live/dead$^-$ (viable) lymphocytes in PBMC and IHL samples after overnight culture with and without bafilomycinA1, Reagent A and MRT68921 dihydrochloride (MitoTracker staining in **Figure S1c;** 14-15 biological replicates). **(d)** The magnitude of the induced $CD69^+CD103^+$ T_{RM} or non-T_{RM} $CD69^-CD103^-$ T cell population as a percentage of total live lymphocytes and **(e)** the viability of total $CD8^+$ T cells after 6 days culture with sequential rhIL-15 then rhTGFβ in the presence of autophagy inhibitors: MRT68921 dihydrochloride (1 μM), and 3-MA (3-Methyladenine, 0.5 mM; 10-32 biological replicates). **(a-b)** Wilcoxon paired t-test between **(a)** untreated and bafA1 treated PBMC and IHL (related to **Figure S1c**; non-significant) and **(b)** untreated and bafA1 treated IHL (related to **Figure S2b**). **(c-e)** Kruskal-Wallis (ANOVA) with Dunn's post hoc test for multiple unpaired comparisons, untreated vs. other treatments (all non-significant; related to **Figure 4g**). $*P < 0.05$; $**P < 0.005$; $****P < 0.0001$.

Sex	Treatment	eAb status	HBV DNA	ALT
F	Untreated	eAb+	127	20
M	Untreated	eAb+	blq	39
M	Untreated	eAb+	436	41
F	Tenofovir	eAb+	blq	27
M	Untreated	eAb+	1072	28
M	Untreated	eAb+	4,400	38
F	Untreated	eAb+	1,100,000	63
F	Untreated	eAb+	blq	nd
M	Untreated	eAb- eAg+ (low)	322	27
M	Untreated	eAb+	blq	19

Table S1. Hepatitis-B virus infected patient characteristics. Related to Figure 3g:
F, Female; M, Male; eAb, anti-hepatitis B virus e antigen antibodies; blq, below level of quantitation; nd, not done.
